# Inflamed Yet Immune-Evasive? A Transcriptomic Meta-Analysis Identifies Conserved Inflammatory, Developmental, and Neuronal Signatures Associated with Polyploid Giant Cancer Cells

**DOI:** 10.3390/ijms27156671

**Published:** 2026-07-26

**Authors:** Olga V. Anatskaya, Alexander E. Vinogradov

**Affiliations:** 1 Institute of Cytology, Russian Academy of Sciences, Saint-Petersburg 194064, Russia; 2 Center of Genetic Reprogramming and Gene Therapy, Institute of Cytology RAS, Saint-Petersburg 194064, Russia

**Keywords:** PGCCs, cancer dormancy, immune evasion, PD-L1, calcium signaling, neuronal mimicry, apoptosis resistance, genome chaos, protein interaction networks, comparative transcriptome analysis

## Abstract

Polyploid giant cancer cells (PGCCs) are increasingly recognized as major drivers of therapy resistance and tumor relapse, yet the conserved molecular programs underlying their persistence remain incompletely defined. To identify genes consistently deregulated across eight independent datasets, we performed an integrative transcriptomic analysis of PGCCs derived from prostate, ovarian, and breast cancers. By focusing on consistently up- or down-regulated genes that were expressed in at least five datasets and showed a concordant direction of expression across more than 70% of datasets and met a significance threshold of adjusted *p* < 0.05, we defined the core regulatory architecture stabilizing the PGCC state under therapeutic stress. Our analysis reveals that PGCCs exhibit a paradoxical ranscriptomic signature consistent with cytolytic activity alongside reduced immune detection. These cells activated pro-inflammatory cytokine and chemokine signaling while simultaneously engaging immune-evasion mechanisms, including PD-L1-associated and virus-like escape programs. Concurrently, PGCCs displayed transcriptional features characteristic of immune-privileged cellular states, including embryonic development, reproductive programs, senescence-associated survival, apoptosis resistance, and deep dormancy marked by coordinated suppression of major housekeeping processes. Notably, PGCCs also activated neuronal differentiation and neurodegeneration-associated pathways, including axon guidance, neurogenesis, and calcium signaling. This neuron-like, calcium-dependent stress adaptation program may further enhance immune privilege and long-term survival capacity. We propose that PGCCs represent an immune-adaptive polyploid survival state in which inflammatory and ontogenetic pathways are repurposed to support immune evasion and tumor persistence. By identifying actionable vulnerabilities within calcium signaling, neuronal mimicry, and checkpoint-associated pathways, this study provides a framework for therapeutic strategies aimed at dismantling the PGCC reservoir and preventing tumor relapse.

## 1. Introduction

A central paradox in cancer biology is that highly aggressive tumors frequently exhibit robust inflammatory and stress-responsive signaling while simultaneously evading immune destruction and persisting under intense therapeutic pressure [1,2]. Although inflammatory activation is classically linked to immune surveillance and tumor elimination, advanced malignancies often sustain chronic cytokine signaling in parallel with immune suppression, checkpoint activation, and profound resistance to cytotoxic stress [3,4]. This apparent contradiction suggests that tumor persistence is not governed by isolated signaling pathways, but rather by integrated cellular states capable of coupling inflammatory activation to immune escape.

Accordingly, cancer progression is increasingly understood as a dynamic process driven not only by genetic alterations, but also by adaptive transitions between alternative cellular states that maximize survival under environmental stress [5,6,7,8,9]. Under conditions such as chemotherapy, hypoxia, inflammation, or immune attack, tumor cells can adopt highly plastic phenotypic configurations characterized by coordinated transcriptional rewiring, stress tolerance, dormancy, and apoptosis resistance [10,11,12]. These observations support an emerging systems-level view in which tumor resilience arises from integrated regulatory programs rather than isolated molecular events [11,13,14,15].

One candidate cellular state capable of reconciling inflammatory activation with immune evasion is the polyploid giant cancer cell (PGCC) state. PGCCs arise across multiple tumor types in response to genotoxic stress, hypoxia, inflammatory signaling, and anticancer therapy [6,16,17,18]. PGCCs are operationally defined and isolated as cancer cells bearing either a single enlarged nucleus or multiple nuclei, with DNA content surpassing the G2/M (4N) threshold as determined by flow cytometry/FACS sorting. Such cells arise preferentially under polyploidizing stress-hypoxia, chemotherapy, or irradiation—and their identity is corroborated through karyotyping and the expression of stemness markers [19,20]. PGCCs are functionally distinct not only from non-PGCCs, the regular-sized, near-diploid tumor cells, but also from their own smaller, diploid progeny, generated through the asymmetric budding division known as neosis [21,22]. Together, these giant cells constitute a dormant-to-active reservoir of treatment resistance, capable of regenerating tumor growth long after conventional therapy has run its course [20].

Once considered aberrant or terminally senescent cells, PGCCs are now recognized as highly viable and plastic cell populations capable of surviving prolonged stress, generating tumor progeny, and promoting relapse [5,19,20,23,24]. Their existence challenges proliferation-centered models of tumor fitness and instead supports the concept that polyploidization functions as a reversible adaptive survival strategy under extreme selection pressure [25,26].

Mechanistically, this adaptive capacity may reflect the reactivation of evolutionarily conserved ontogenetic programs [13,15,27,28,29]. High-plasticity states resembling embryogenesis, reproductive biology, dormancy, senescence-associated survival, neuronal differentiation, and immune-privileged tissue programs share common features of stress resistance, developmental flexibility, and reduced immune vulnerability [30,31,32]. Tumor cells may exploit these deeply conserved programs to maintain long-term survival in hostile microenvironments [27,29,33,34].

Polyploidization may provide the epigenetic and transcriptional framework enabling this transition by increasing regulatory plasticity and relaxing constraints that normally segregate developmental, inflammatory, and stress-response pathways in diploid proliferating cells [29,35,36,37]. Through this systems-level integration, PGCCs may simultaneously activate inflammatory signaling and immune-evasion programs, thereby resolving the paradoxical coexistence of immune activation and reduced immune visibility [38,39].

Despite increasing recognition of PGCCs as drivers of therapy resistance and relapse, the conserved molecular logic underlying this state remains poorly defined. Here, using an integrative transcriptomic meta-analysis across multiple cancer types, we identify a recurrent, conserved gene-expression signature whose co-enrichment pattern is consistent with a systems-level architecture that could support PGCC survival under severe stress. Our data show that genes and pathways associated with inflammatory activity and genes and pathways associated with immune evasion are co-up-regulated in PGCCs and connected through shared hub regulators within the reconstructed protein–protein interaction network. We interpret this pattern as raising the hypothesis that PGCCs organize inflammatory activity into a self-reinforcing network functionally coupled to immune evasion, while noting that a functional or causal link between these co-expressed programs is not established by the present transcriptomic data. Its recurrence across tumor types is consistent with the view that the PGCC state reflects a conserved transcriptional survival strategy rather than a pathological byproduct and nominates its core hub regulators as candidate targets for future therapeutic investigation.

## 2. Results

To define the transcriptional architecture underlying PGCC stress tolerance, we integrated transcriptomic datasets from multiple studies comparing PGCCs and diploid cancer cells of the same origin across prostate, ovarian, and breast cancers. Despite inter-study variability, a conserved transcriptional signature emerged. Focusing on consistently dysregulated genes, we identified a shared PGCC program enriched in highly connected hub genes within protein–protein interaction networks, including 198 up-regulated and 248 down-regulated nodes. The lists of PGCC up- and down-regulated genes with integrated average fold (log2); *p*-values, and FDR are presented in Supplementary Materials Tables S1 and S2. Supplementary Materials Figure S1A,B present heatmaps with integrative *p*-values for all consistently up- and down-regulated genes from all eight databases. Supplementary Materials Figures S2 and S3 present enrichment of all PGCC-up-regulated and down-regulated genes in GO biological processes and KEGG, Reactome, and Wiki signaling pathways provided by Metascape.

Functional enrichment analysis resolved distinct but convergent programs, whose integration through shared regulatory nodes defines the PGCC immune-evasive state. Together, these results delineate a core transcriptional pattern that distinguishes PGCCs from diploid cancer cells and encodes their stress tolerance and immune evasion at the systems level.

### 2.1. Cytolytic Yet Immunologically Invisible? PGCCs Co-Express Inflammatory, Antiviral, and Immunosuppressive-Associated Gene Modules

Transcriptomic analysis shows that PGCCs simultaneously express gene modules associated with cytolytic inflammatory activity and gene modules associated, in other contexts, with immune escape. This co-expression pattern distinguishes PGCCs from diploid cancer cells in our dataset. Our results show that PGCCs exhibit elevated expression of a broad spectrum of immunity-related gene modules, including processes that are typically considered functionally opposed, such as inflammation-associated and immune-evasion-associated signatures. We interpret this combination as raising the hypothesis that PGCCs may function as cytolytic yet immunologically “invisible” cancer cells; direct functional evidence for aggressive behavior coupled with escape from immune surveillance was not obtained in this study [40]. The inflammatory and antiviral programs underlying this paradoxical state are described in detail below.

### 2.2. The Activation of Inflammatory and Antiviral Programs

**Figure 1:**
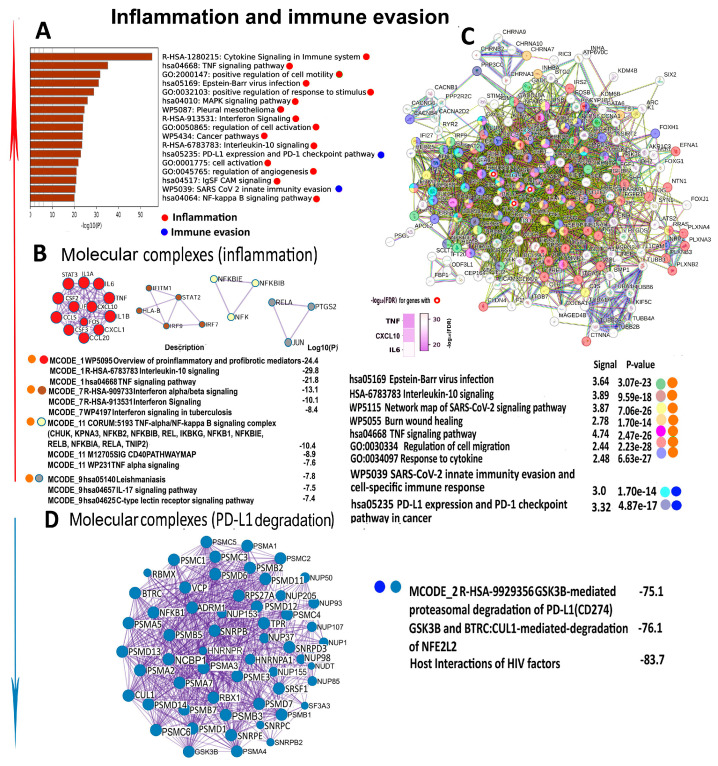
Immune-inflammatory signaling and PD-L1 regulatory networks in PGCCs. (**A**) Functional enrichment analysis of PGCC up-regulated genes associated with immune regulation. The most significantly enriched pathways and biological processes include cytokine signaling, TNF, IFN, IL-10, IL-17, NF-κB, and MAPK signaling, as well as Epstein–Barr virus infection, mesothelioma-related pathways, cell activation, angiogenesis, cell motility, and immune checkpoint regulation. Bars represent enrichment significance expressed as *−log10(P)*. (**B**,**C**) MCODE-based identification of highly interconnected molecular complexes within the PGCCs up-regulated inflammatory network. Distinct modules are enriched for inflammatory and profibrotic mediators, TNF, IL-10, IL-17, interferon signaling, NF-κB activation, and pathogen-associated immune pathways. Representative hub genes include IL1A, IL1B, IL6, TNF, CXCL1, CXCL5, CXCL10, CSF2, CSF3, FOS, STAT3, RELA, NFKB1, NFKBIA, and IRF family members, highlighting the establishment of a robust inflammatory signaling state in PGCCs. (**C**) Global PPIN for immune-associated PGCCs up-regulated genes. PPIN for PGCC-up-regulated genes enriched for various gene modules. Nodes represent proteins encoding for genes, and pie-chart sectors inside gene symbols indicate participation in significantly enriched Gene Ontology (GO) biological processes and signaling pathways from Reactome, KEGG, and Wikipathways databases. Node color coding within the network is shown adjacent to the column displaying *p*-values. Network topology reveals extensive connectivity between inflammatory cytokines, stress-response regulators, transcription factors, and signaling mediators. Enrichment analysis of the network confirms activation of pathways linked to Epstein–Barr virus infection, IL-10 signaling, TNF signaling, wound healing, cytokine responses, cell migration, SARS-CoV-2 immune evasion pathways, and PD-L1/PD-1 checkpoint regulation. Genes participating in more than 60% of all marked gene modules (for this figure, it is eight) are marked with red-white points in the middle of gene symbols and their FDR integrative through all eight databases are presented as one column heat map. (**D**) Molecular complex comprised by PGCCs down-regulated protein encoding genes that are associated with PD-L1 degradation. A prominent module consists of proteasomal, ubiquitination, RNA-processing, and nuclear pore components, including PSMA/PSMB/PSMC/PSMD family members, RBX1, CUL1, BTRC, GSK3B, VCP, and nucleoporins. Functional enrichment identifies GSK3β- and βTrCP/CUL1-mediated proteasomal degradation of PD-L1 (CD274) as the dominant network feature, suggesting enhanced post-translational regulation of immune checkpoint signaling in PGCCs. Overall, the figure illustrates a paradoxical PGCC phenotype characterized by simultaneous activation of inflammatory and stress-response programs together with regulatory mechanisms that promote immune evasion through modulation of PD-L1 signaling and checkpoint-associated pathways.

The Metascape and STRING analyses revealed strong enrichment of cytokine- and interleukin-mediated signaling pathways, including NF-κB, TNF, AP-1, interferon responses, and antiviral defense programs (Figure 1A–C, Supplementary Materials Figure S2) The MCODE analysis further identified highly interconnected protein complexes associated with TNF signaling, cytokine-mediated immunity, antigen processing and presentation, and pro-inflammatory regulation (Figure 1B).

These findings are consistent with prior reports linking PGCCs to cytokine-driven tumor progression and therapy resistance [41,42,43], and suggest that PGCCs sustain a cytokine-rich inflammatory niche. Concordantly, Pienta et al. characterized PGCCs as a reversible, drug-tolerant population capable of surviving lethal therapeutic stress and re-seeding tumor growth driven in part by cytokine-mediated survival signaling [44].

Consistent with the established role of TNF and NF-κB signaling in promoting epithelial-mesenchymal transition, migration, and immune invasion [45], our data revealed striking activation of gene modules associated with cellular motility and locomotion (Figure 1A, Supplementary Materials Figure S2). This corroborates prior observations of enhanced metastatic potential in PGCCs and suggests that this migratory phenotype is evolutionarily conserved across diverse PGCC types [24,46]).

PGCCs also displayed strong activation of interferon and antiviral response pathways, including signatures linked to COVID-19, Epstein–Barr virus, human papillomavirus, and HTLV-1 infection (Figure 1A,C). Up-regulated genes encompassed innate pathogen-sensing receptors TLR2, DDX58, and DHX58 alongside interferon-stimulated genes ISG15, IFI27, IFI6, OAS1, and MX1, indicative of active innate immune sensing and antiviral defense (Supplementary Materials Table S1).

Collectively, these observations indicate that PGCCs possess a distinctive pro-inflammatory and antiviral transcriptional landscape relative to diploid cancer cells; whether this landscape supports tumor-promoting plasticity, as opposed to reflecting it, cannot be determined from expression data alone.

### 2.3. PD-L1-Associated and Antiviral-Associated Signaling: Candidate Contributors to Immune Evasion

Transcriptomic analysis showed that PGCCs up-regulate PD-L1-checkpoint-associated gene expression and a gene module annotated to immune evasion in SARS-CoV-2 infection; we interpret the co-occurrence of these signatures as consistent with, though not proof of, a multilayered immune-evasion architecture. The up-regulated PD-1/PD-L1-associated regulatory gene set in PGCCs includes multiple well-characterized regulators of PD-L1/CD274, including NF-κB components (RELA, NFKB2, NFKBIE, NFKBIB), stress-response transcription factors (FOS, JUN), and mediators of inflammation and survival (STAT3, ZAP70, EGF, HIF1A, CD4, CD28, TLR2, IRF1) (Figure 1A–C, Supplementary Materials Table S1).

This transcriptional landscape is consistent with established mechanisms governing PD-L1 expression [1]. Among the down-regulated genes, MCODE clustering identified a giant molecular complex unifying more than 60 genes (Figure 1D). This complex is enriched for the Reactome pathway “GSK3B-mediated proteasomal degradation of PD-L1” (R-HSA-9929356) with very high statistical significance *p* < 10^−75^. Notably, GSK3B itself was markedly down-regulated, a pattern consistent with, though not direct evidence of, reduced PD-L1 turnover and stabilization of the checkpoint axis in PGCCs (Figure 1D, Supplementary Materials Table S2).

PGCC-induced genes also exhibited enrichment of the pathway “Innate immune evasion of SARS-CoV-2 and cell-type-specific immune response” (Figure 1A). Given that PGCCs are not SARS-CoV-2-infected; this transcriptional signature likely reflects the activation of a conserved antiviral stress program rather than a bona fide infection. This interpretation is reinforced by the pronounced up-regulation of interferon-stimulated genes in PGCCs, including ISG15, OAS1, MX1, and members of the IFIT and IRF families (Figure 1A,C, Supplementary Materials Table S1). This signature is increasingly recognized as a hallmark of genomic instability in cancer [47].

Altogether, these observations suggest that PGCCs may integrate stress-induced innate immune pathways to paradoxically promote immune evasion via multistep molecular circuitry. Based on these observations, it is quite reasonable to suggest that cytosolic DNA arising from genomic instability may activate interferon-stimulated gene (ISG) programs [48], which in turn promote IRF- and STAT-dependent transcription of PD-L1 [49], outlining a speculative mechanistic model linking genomic instability, interferon-stimulated gene expression, and checkpoint-associated gene expression that remains to be tested functionally.

Beyond checkpoint-associated signaling, PGCCs also express physiological gene programs typically associated with embryonic development and stress adaptation. In other biological contexts, each of these programs has been individually linked to suppression of immune recognition; whether they jointly reinforce an immune-evasion architecture in PGCCs, as opposed to being independently co-activated, is a hypothesis raised by their co-expression rather than a conclusion established here.

### 2.4. Physiological Programs Further Promoting Immune Evasion

Beyond this checkpoint-mediated immune escape and programs of viral immunity evasion, PGCCs appear to leverage a broader arsenal of strategies promoting apparent immune evasion, including development, germline/reproductive reprogramming, senescence, dormancy, neuronality, and apoptosis resistance. Below we provide a detailed description of all of them.

### 2.5. Senescence, Development, and Germline/Reproductive Reprogramming

**Figure 2:**
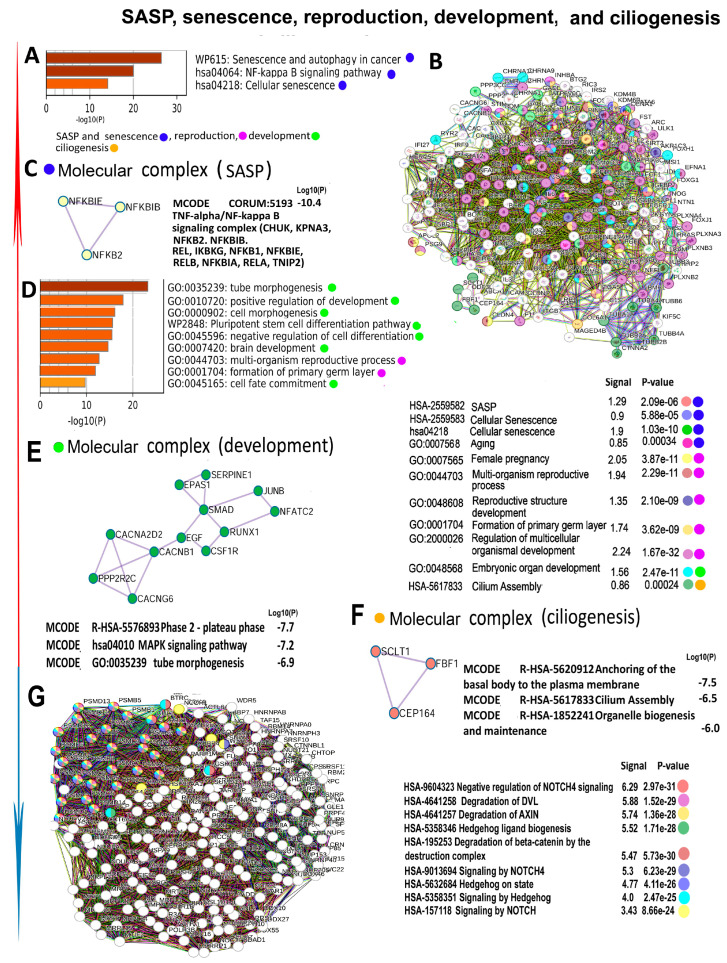
Activation of senescence-, development-, reproduction-, and ciliogenesis-associated gene programs in polyploid giant cancer cells (PGCCs). This multi-panel figure summarizes functional enrichment and molecular interaction landscapes underlying senescence-associated secretory phenotype (SASP) programs and their functional coupling with cellular senescence, developmental regulation, reproductive biology, and ciliogenesis. (**A**) Functional enrichment analysis of up-regulated genes identifies significant activation of pathways associated with cellular senescence, senescence-associated secretory phenotype (SASP), autophagy in cancer, and NF-κB signaling. Bars represent enrichment significance expressed as *−log10(P)*. (**B**) Integrated protein–protein interaction network of genes involved in senescence, development, reproduction, and ciliogenesis, demonstrating extensive functional connectivity among these biological programs. Representative enriched pathways and Gene Ontology (GO) terms are shown alongside their enrichment significance. Node color coding within the network is shown adjacent to the column displaying *p*-values. (**C**) MCODE analysis identifies a highly interconnected SASP-associated molecular complex centered on the canonical TNFα/NF-κB signaling module, including NFKB1, NFKB2, NFKBIA, NFKBIB, and related regulatory proteins, highlighting activation of inflammatory senescence signaling. (**D**) Enrichment analysis of development- and reproduction-related genes reveals significant overrepresentation of pathways governing morphogenesis, stem cell differentiation, cell fate determination, embryonic development, and germ layer formation. (**E**) Development-associated molecular complex enriched for regulators of morphogenesis and differentiation, including SMAD, RUNX1, EGF, NFATC2, EPAS1, and SERPINE1, with enrichment for MAPK signaling, tube morphogenesis, and developmental progression. (**F**) Ciliogenesis-associated molecular complex comprising CEP164, FBF1, and SCLT1, key components of basal body anchoring and primary cilium assembly. Associated pathways indicate activation of ciliogenesis together. (**G**) Global interaction network for PGCCs down-regulated genes integrating all identified molecular complexes, illustrating partial down-regulation of NOTCH4 and Hedgehog signaling networks related to programs of development. Node color coding within the network is shown adjacent to the column displaying *p*-values.

Three programs presented by senescence, development, and germline/reproductive reprogramming emerge as particularly synergistic drivers of PGCC persistence, each independently linked to immune evasion [1,50]. PGCC transcriptional profiles revealed a pronounced senescence-associated secretory phenotype (SASP) signature (Figure 2A–C), a state known to promote immune evasion through induction of immune-checkpoint pathways, including PD-L1, and establishment of an immunosuppressive microenvironment [50,51,52,53]. The association between PGCCs and SASP was previously identified in several studies [54,55,56].

Our data further expanded the repertoire of SASP-associated signaling pathways and regulatory molecules in PGCCs. Metascape found strong enrichment for SASP-related signaling pathways, particularly NF-κB signaling (hsa04064, Signal 3.1, *p* < 10^−16^) (Figure 2A), indicating activation of a central inflammatory and stress-adaptive regulatory program. This enrichment included key NF-κB components, composing the MCODE molecular complex presented by NFKB2, NFKBIB, and NFKB2 (Figure 2A). Consistently, the enrichment of the “Senescence and autophagy in cancer” and “Cellular senescence” pathways further supported the presence of a senescence-like cellular state (Figure 2A). STRING analysis additionally revealed significant enrichment of the terms “Aging” (GO:0007568), “SASP,” and “Cellular senescence” (Figure 2B). The up-regulation of canonical senescence-associated genes, like CDKN1A (p21), GADD45A/B, and BTG2 (Figure 2A,B and Supplementary Materials Table S1) further confirms the association between PGCCs and senescence. Notably, however, several classical markers of irreversible senescence, including CDKN1B (p27), CDKN2C (p18), and XRCC5/XRCC6 were consistently down-regulated across datasets (Supplementary Materials Table S2). In addition, the key regulator of senescence CDKN2A (p16) did not show meaningful difference. These data indicate that PGCCs occupy a non-canonical, potentially reversible, senescence-like state rather than terminal senescence. Together, these observations indicate that PGCCs co-express a SASP-associated inflammatory signature with markers of reversible, rather than terminal, senescence; based on roles reported for these programs elsewhere, we hypothesize that this configuration could contribute to immune suppression and long-term tumor persistence, though this was not directly tested.

Beyond the activation of SASP-related programs, our data revealed co-enrichment of pathways associated with embryonic development, germline (i.e., formation of primary germ layer), and reproductive reprogramming. Metascape identified nine gene modules related to development and reproduction (Figure 2C), and a molecular complex “Tube morphogenesis” (Figure 2C). Consistent with this, STRING analysis determined several gene modules related to reproduction and embryonic development (Figure 2B). These pathways warrant attention because by activating developmentally immune-privileged states, these programs can promote immune evasion through suppression of cytotoxic immunity, destabilization of antigenicity, and enforcement of checkpoint dominance [57,58].

The activation of pro-embryonic and pro-reproductive programs in PGCC was previously observed in many studies and currently they are considered as hallmarks of PGCCs [21,59,60,61,62,63]. These studies suggested that PGCCs can activate evolutionarily conserved archaic embryonic programs and germ-layer differentiation that can be de-repressed for oncogenesis [21,64,65]. Our data also found the activation of canonical regulators of developmental plasticity, like SHH, NOTCH1/3, JAG1, HES1, and TGFβ/SMAD signaling (Supplementary Materials Table S1) promoting dedifferentiation while reducing differentiation-associated antigenicity and sustaining immune-privileged cellular states [66]. In accordance with the activation of programs of development, MCODE revealed a molecular complex related to ciliogenesis enhancing signaling of development (Figure 2D) whereas STRING analysis identified significant enrichment of the pathway “Cilium assembly” (Figure 2B) confirming prior evidence of prominent cilia in PGCCs and differentiated polyploid cells [67,68,69]. Cilium assembly enrichment is functionally significant given the established role of this organelle in potentiating development signaling and suppressing anti-tumor immunity [70].

The activation of gene modules related to development was balanced by the partial down-regulation of Hedgehog signaling, Hedgehog-associated signaling via Gli 1-3 (Figure 2G), and a HOTCH4 branch of NOTCH signaling (Figure 2G). The concurrent down-regulation of these pathways is very consistent with a tumor PGCC dormant state because their high activity has been linked to PGCCs reactivation [18,71]. Collectively, the transcriptional landscape of PGCCs shows a paradoxical coexistence of an embryonic reprogramming signature, which we interpret as a candidate immune-evasion-associated program, with coordinated down-regulation of Hedgehog and NOTCH4, two developmentally interconnected pathways. Their reactivation has been linked to PGCC awakening, thereby defining a molecular dormancy signature that is simultaneously immunologically shielded with the potential for proliferative reactivation. This complex configuration is further consolidated by dormancy that is an actively enforced state of proliferative arrest that minimizes immunogenic exposure.

### 2.6. PGCCs Implement an Active Dormancy Program Linked to Immune Evasion

**Figure 3:**
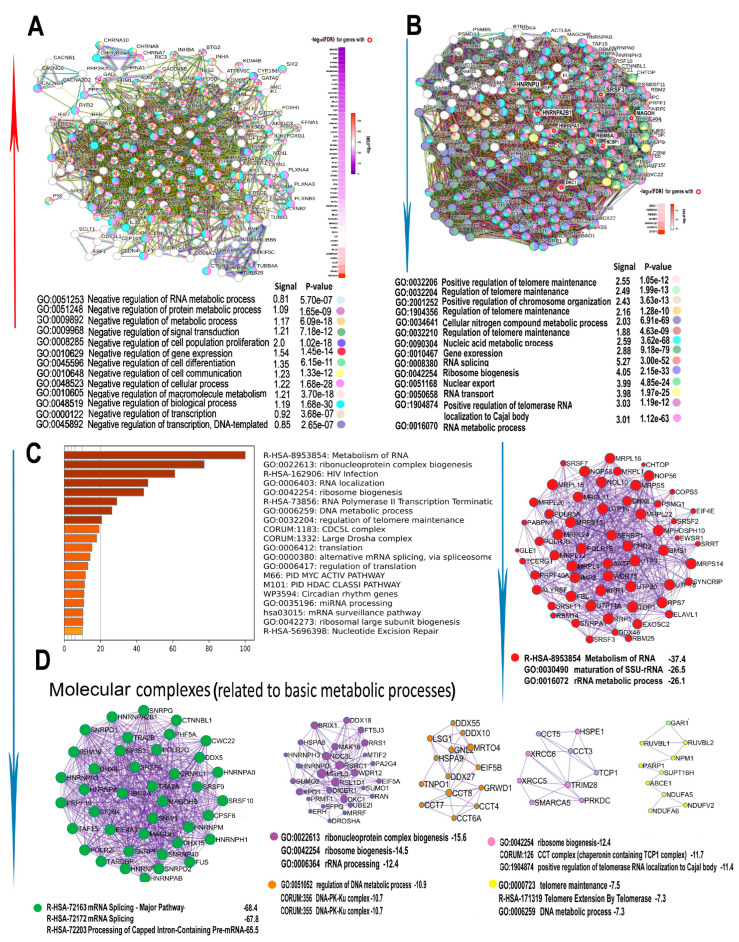
Functional network architecture of genes participating in dormancy in PGCCs. (**A**) Global PPIN for PGCC-up-regulated genes governing dormancy enriched for biological processes related to active negative regulation of basic (house-keeping) processes in a cell. Nodes represent proteins encoding for genes and pie-chart sectors inside gene symbols indicate participation in significantly enriched Gene Ontology (GO) biological processes. Here and in (**B**), node color coding within the network is shown adjacent to the column displaying *p*-values. Genes participating in more than 60% of all marked gene modules (for this figure, it is eight) are marked with red-white points in the middle of gene symbols and their FDR integrative through all eight databases are presented as one column heat map. The network is predominantly enriched in pathways involved in negative regulation of RNA metabolism, protein metabolism, cellular signaling, transcription, cell proliferation, differentiation, communication, and macromolecule biosynthetic processes, indicating a broad suppression of regulatory and homeostatic cellular functions in PGCCs. Bars represent enrichment significance expressed as *−log10(P)*. (**B**) Functional interaction network of proteins encoding for the PGCCs down-regulated genes. All designations characterizing the nodes are the same as in Figure 3A. The network is associated with RNA metabolism, telomere maintenance, chromosome organization, RNA processing, ribosome biogenesis, nucleocytoplasmic transport, and RNA splicing. Highly connected hub genes, including *HNRNPU*, *DKC1*, *NCBP1*, and other RNA-processing factors, occupy central positions within the network, suggesting coordinated repression of RNA maturation and telomere-associated regulatory programs. Colored dots indicate major functional categories: inflammation (orange), migration (green), immune evasion (blue), and cancer-related pathways (gray). Bars represent enrichment significance expressed as *−log10(P)*. (**C**) Pathway enrichment analysis of the down-regulated gene set. Bar lengths indicate the relative enrichment significance of Reactome, Gene Ontology, CORUM, KEGG, WikiPathways, and Human Phenotype Ontology terms. The most significantly affected pathways include RNA metabolism, ribonucleoprotein complex biogenesis, RNA localization, ribosome biogenesis, RNA polymerase II transcription termination, DNA metabolic processes, telomere maintenance, mRNA processing and surveillance, translation, and nucleotide excision repair, highlighting extensive impairment of fundamental gene-expression machinery. Genes participating in more than eight gene modules are marked with orange/white points in the middle of gene symbols, and their FDR integrative through all eight databases is shown as one column heat map. (**D**) Protein–protein interaction subnetworks illustrating major molecular complexes obtained with MCODE that are suppressed in PGCCs. Distinct clusters correspond to the spliceosome and mRNA-splicing machinery (green), ribonucleoprotein and ribosome-biogenesis complexes (purple), DNA repair and DNA-dependent protein kinase (DNA-PK) complexes (orange), CCT chaperonin-containing TCP1 complex and telomerase-associated factors (pink), and telomere maintenance and mitochondrial regulatory modules (yellow). Values shown beneath each cluster represent integrated pathway activity scores, with negative values indicating coordinated down-regulation of the corresponding molecular systems. Collectively, these analyses demonstrate that PGCCs exhibit widespread repression of RNA processing, ribosome biogenesis, DNA repair, and telomere maintenance pathways, consistent with a profound remodeling of core cellular metabolic and gene-expression programs.

Our data support the view that polyploid giant cancer cells (PGCCs) represent a stress-induced dormant state that emerges following genotoxic insults or microenvironmental stress [16,72]. Tumor dormancy and immune evasion are closely interconnected processes. Dormant cancer cells can persist for prolonged periods by adapting to an immunosuppressive microenvironment and reducing their immunological visibility, including through PD-L1-associated pathways, thereby contributing to disease recurrence [25,32]. Bioinformatic analysis indicated that gene expression patterns consistent with an actively regulated dormant state are present in PGCCs. The STRING analysis revealed significant enrichment of the GO category “Negative Regulation of Cell Population Proliferation” (GO:0008285), indicating that PGCC dormancy represents a coordinated transcriptional program rather than a passive loss of proliferative capacity (Figure 3A, Supplementary Materials Table S1). As shown in Figure 3A, this biological process involves approximately 30 genes (symbol sectors highlighted in blue). Importantly, these include conserved regulators of cell-cycle arrest, such as CDKN1A (p21), BTG2, GADD45A/B, and LATS2 (Figure 3A, Supplementary Materials Table S1), which promote G0/G1 cell-cycle arrest under stress conditions [73,74,75]. Antiproliferative transcription factors, including KLF2, KLF6, EPAS1, INHBA, and others (Figure 3A, Supplementary Materials Table S1), further reinforce cellular quiescence by suppressing cell-cycle genes and activating stress-adaptive transcriptional programs associated with cellular tolerance and survival [76,77,78,79]. Accordingly, activation of MAPK13 (Supplementary Materials Table S1), together with suppression of c-Myc (Supplementary Materials Table S2) and MYC-regulated target genes (M66: PID MYC ACTIVE PATHWAY, Figure 3B), indicates a transition toward a metabolically economical, drug-tolerant diapause-like state [80]. Another important feature of the PGCC transcriptome supporting dormancy is the activation of a broad spectrum of pathways involved in the negative regulation of metabolic processes and signaling cascades, including those governing gene expression, metabolism, signal transduction, proliferation, and differentiation (Figure 3A).

The principal regulators that were consistently activated across all eight datasets with extremely high statistical significance (integrative *p* < 10^−30^) and participated in the largest number of fundamental biological processes included CDKN1A (p21), the hypoxia-responsive factor EPAS1 (HIF-2α), the stress-responsive transcription factor ATF3, the pro-fibrotic TGF-β family regulator INHBA, cancer stem cell-associated markers CD44, KLF2, and GATA6, as well as the anti-apoptotic regulator BCL6 (Figure 3A, Supplementary Materials Table S1). Collectively, these regulators define a conserved molecular architecture of PGCC dormancy that integrates cell-cycle arrest, stress adaptation, immune modulation, development, metabolic reprogramming, and survival signaling. Overall, our data revealed 53 dormancy-related master regulators that could potentially provide a basis for developing therapeutic strategies aimed at suppressing dormancy in cells (Figure 3A).

In accordance with active negative regulation of housekeeping processes, demonstrated by the up-regulated genes, the down-regulated genes were enriched in ample gene modules related to protein synthesis, transcription, translation, gene expression, and circadian rhythm (Figure 3B). Moreover, MCODE clustering discovered very tight complexes related to mRNA splicing and metabolism (green and red), unifying 37 and 53 genes and several complexes implicated in protein synthesis, DNA metabolism, and telomere maintenance (Figure 3C). The global network of the down-regulated genes further supports dormant state and metabolic suppression of PGCC. It is evident from the enrichment in gene modules related to RNA splicing, ribosome biogenesis, and telomere maintenance (Figure 3D). The main regulators are presented at the heatmap at Figure 3D. Collectively, these observations are consistent with PGCC dormancy reflecting a regulated transcriptional program that couples cell-cycle-arrest and metabolic-suppression signatures; whether this program actively enables immune evasion and long-term persistence, rather than merely co-occurring with them, remains a hypothesis, and the identified regulatory hub genes represent candidate rather than validated therapeutic targets.

### 2.7. Apoptosis Resistance

**Figure 4:**
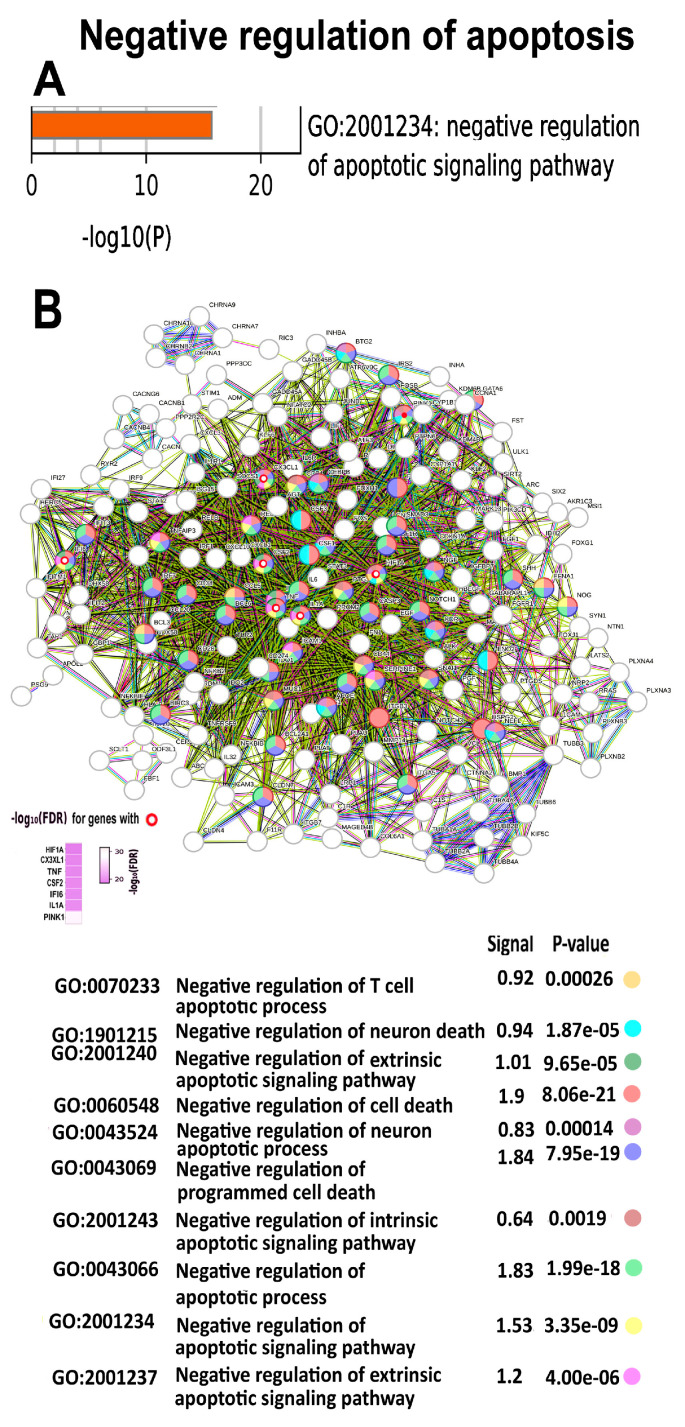
Anti-apoptotic signaling network in polyploid giant cancer cells (PGCCs). (**A**) The Metascape GO enrichment analysis demonstrating significant activation of the biological process “Negative regulation of apoptotic signaling pathway” (GO:2001234) among genes up-regulated in PGCCs. The horizontal bar represents the enrichment significance expressed as *−log10(P)*, indicating a robust suppression of apoptosis-related signaling. (**B**) Functional interaction network of genes contributing to the negative regulation of apoptosis in PGCCs. Nodes represent genes, while edges indicate validated functional associations. Colored sectors in gene symbols denote the participation of individual genes in multiple anti-apoptotic GO categories, including negative regulation of apoptotic signaling, programmed cell death, intrinsic and extrinsic apoptotic pathways, neuron death, and T-cell apoptosis. Node color coding within the network is shown adjacent to the column displaying *p*-values. Genes participating in more than 60% of all marked gene modules (for this figure, it is six) are marked with red-white points in the middle of gene symbols and their FDR integrative through all eight databases are presented as one column heatmap. Collectively, the network reveals a highly interconnected survival program characterized by simultaneous suppression of intrinsic and extrinsic apoptotic pathways, inhibition of programmed cell death, and reinforcement of pro-survival signaling. These findings support the concept that PGCCs acquire a robust apoptosis-resistant phenotype, promoting cellular persistence under stress and facilitating long-term survival, dormancy, and potential tumor recurrence.

One more important feature of PGCCs is an apoptosis-resistant state [43]. Tumor cells that attenuate apoptotic execution pathways gain a decisive survival advantage, allowing them to evade immune surveillance and persist under sustained immune pressure [81]. The Metascape analysis identified the enrichment of PGCCs up-regulated genes for “Negative regulation of apoptotic signaling pathway” (Figure 4A), whereas the STRING analysis uncovered the enrichment of 10 GO biological processes suppressing extrinsing and intrinsing apoptosis and cell death (Figure 4B). Collectively, these observations indicate that PGCCs show multi-layered down-regulation of gene modules associated with both extrinsic and intrinsic apoptotic pathways; based on the roles of these pathways described elsewhere, we hypothesize this signature could confer a survival advantage contributing to PGCC persistence, though this was not functionally tested here.

### 2.8. Neuronality and Neurodegeneration

**Figure 5:**
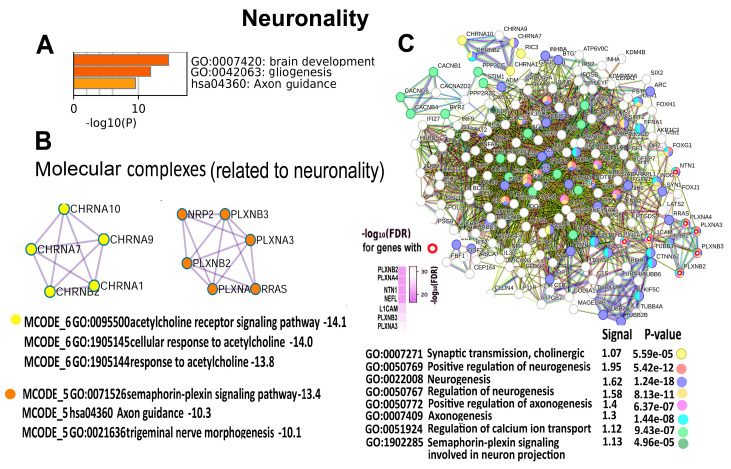
Neuronality-associated transcriptional program and interaction network in PGCCs. (**A**) Functional enrichment analysis of neuronality-related genes up-regulated in polyploid giant cancer cells (PGCCs). Bar lengths represent enrichment significance expressed as −log10(*p*-value). (**B**) Molecular complexes associated with neuronal differentiation and neural signaling identified by MCODE analysis. Two highly interconnected modules were detected. The first module is enriched for acetylcholine receptor signaling and cholinergic response pathways, including *CHRNA1*, *CHRNA7*, *CHRNA9*, *CHRNA10*, and *CHRNB2*. The second module is enriched for semaphorin–plexin signaling, axon guidance, and neuronal morphogenesis, comprising *NRP2*, *PLXNA2, PLXNA3*, *PLXNB2*, *PLXNB3*, and *RRAS*. Enriched Gene Ontology and pathway annotations are indicated below each module together with their enrichment scores (−log10(FDR). (**C**) STRING protein–protein interaction network of neuronality-associated genes. Nodes representing proteins and edges indicate experimentally validated or predicted functional interactions. Colored sectors identify genes belonging to significantly enriched neuronal processes, including cholinergic synaptic transmission, neurogenesis, positive regulation of neurogenesis, regulation of neurogenesis, axonogenesis, positive regulation of axonogenesis, regulation of calcium ion transport, and semaphorin–plexin signaling involved in neuron projection guidance. Node color coding within the network is shown adjacent to the column displaying *p*-values. Genes participating in more than 60% of all marked gene modules (for this figure, it is five) are marked with red-white points in the middle of gene symbols and their FDR integrative through all eight databases are presented as one column heat map. The network reveals extensive integration of developmental neuronal signaling, axon guidance machinery, calcium signaling, and cholinergic communication pathways within the PGCC transcriptome. Collectively, these findings demonstrate activation of a coordinated neuronality-associated transcriptional program in PGCCs, characterized by the induction of neurodevelopmental, axon-guidance, and cholinergic signaling networks. This neuronal-like phenotype may contribute to cellular plasticity, stress adaptation, tumor progression, and interactions with the tumor microenvironment, thereby representing a potential source of therapeutic vulnerabilities in dormant and treatment-resistant cancer cell populations.

The final and perhaps most unexpected component of the immune-evasion architecture identified in PGCCs is the activation of neuronal programs, an emerging hallmark of tumor adaptation that enables malignant cells to communicate with, remodel, and survive within their microenvironment [82]. Here, neuronal programs refer to the reactivation of gene networks that normally govern neuronal development, axon guidance, synaptic communication, and calcium signaling, rather than the acquisition of a neuronal identity. Accordingly, Metascape identified gene modules associated with axon guidance, brain development, and gliogenesis (Figure 5A), while MCODE clustering revealed molecular complexes involved in cholinergic signaling and axon guidance mediated by members of the cholinergic receptor (CHRN) family and semaphorin–plexin signaling networks governed by plexins (PLXNs) (Figure 5B). STRING analysis further highlighted plexins as the principal hubs of the pro-neuronal protein–protein interaction network, participating in six of eight functional modules (Figure 5C). Beyond their established role in neural development, semaphorin–plexin pathways orchestrate immune-cell migration, stromal organization, and tissue architecture, thereby contributing to the formation of immune-excluded niches that physically restrict lymphocyte infiltration [83]. Consistently, STRING analysis also revealed significant enrichment of neuronal signaling, axonogenesis, and calcium-regulatory pathways (Figure 5C). Collectively, these findings support the concept of cancer–neuron mimicry, whereby tumor cells selectively appropriate neuronal signaling pathways to enhance fitness within hostile microenvironments rather than adopting a neuronal phenotype. Among these pathways, calcium signaling occupies a central position. As a master regulator of cellular communication, cytoskeletal dynamics, migration, and secretion, calcium signaling has emerged as a key driver of adaptive tumor plasticity and immune suppression [84].

Strikingly, the convergence of these neuronal-associated features produces a transcriptional signature that resembles gene-expression patterns reported in neuronal injury and neurodegeneration, a pattern consistent with, though not proof of, an association between polyploidy and neurodegeneration-associated states [85]. In PGCCs, calcium-dependent signaling mediated by PPP3CC/NFATC2, RYR2, and STIM1 (Figure 5C; Supplementary Materials Table S1) is predicted to sustain persistent Ca^2+^ flux, promoting large-scale transcriptional remodeling and increasing cellular plasticity under immune and therapeutic pressure [86,87,88].

Notably, the coordinated activation of neuronal signaling extends beyond functional mimicry and culminates in a distinct transcriptional state reminiscent of neurodegenerative processes. We use this term to describe a molecular profile that resembles chronically stressed or injured neurons, rather than neurodegenerative disease itself. This state emerges through the convergence of persistent calcium dysregulation, chronic inflammatory signaling, and extensive cytoskeletal remodeling—processes that collectively characterize neuronal injury and drive adaptive transcriptional reprogramming [89]. The simultaneous activation of inflammatory and neuronal pathways therefore represents a coherent stress-adaptation program rather than two independent biological processes.

There is enrichment of pathways related to brain development and gliogenesis (Figure 5A), together with the reactivation of developmental regulators including SHH, NOTCH1/3, FOXG1, MSI1, PROM1, and L1CAM (Supplementary Materials Table S1). Consistent with our previous transcriptomic analysis [14], PGCC-up-regulated genes were also significantly enriched for the GO term “positive regulation of multicellular organismal processes” (*p* < 10^−38^; Supplementary Materials Figure S4A). Functional annotation of this gene set showed that it predominantly comprises regulatory pathways involved in neuronal signaling, immune responses, stress adaptation, cell migration, and developmental processes (Supplementary Materials Figure S4B), supporting the coordinated nature of the transcriptional reprogramming observed in PGCCs. We hypothesize that this neuronal-associated transcriptional program represents a coordinated adaptive response contributing to structural resilience, altered interactions with the tumor microenvironment, and transcriptional flexibility under therapeutic stress; however, this interpretation remains speculative and was not tested functionally [90].

Taken together, our observations are consistent with, and motivate, a speculative hierarchical model of neuronal adaptation in PGCCs that remains to be tested. In this model, reactivation of neuronal developmental programs would provide the molecular framework for cancer–neuron mimicry, which we hypothesize could allow tumor cells to exploit neuronal communication pathways for immune evasion and environmental adaptation. Sustained calcium signaling and chronic inflammatory activation might subsequently consolidate this response into a neurodegeneration stress state, potentially preserving cellular plasticity and promoting long-term survival. We propose, as a speculative model requiring functional validation, that neuronal-associated gene expression, cancer-neuron mimicry, and gene signature reminiscent of neurodegenerative processes represent successive and interconnected layers of an adaptive architecture that could contribute to PGCCs evading antitumor immunity, persisting during therapy, and driving tumor recurrence; this model is not directly demonstrated by the present transcriptomic data.

### 2.9. Whole-Genome Doubling Drives Convergent Activation of Neuronal-like Plasticity and Suppression of Pro-Apoptotic, Metabolic, and Immunity-Related Programs in PGCCs and in Non-Giant Polyploid Cancer Cells (WGD+ Cells)

It is well established that PGCCs and WGD+ cells differ substantially in their physiology. PGCCs are dormant, non-proliferating cells with moderate metabolic activity [18], whereas non-giant polyploid cancer cells are non-dormant, proliferative, and metabolically more active than PGCCs [91]. This physiological difference is also evident from the fact that the overall similarity distance between the expression folds of PGCC/diploid cancer cells and WGD+/WGD− cancer cells is slightly negative (Pearson r = −0.31, Spearman r = −0.25 for correlation of log2 fold changes; *p* < 10^−20^ for both), consistent with this previously described distinction in their nature (Vinogradov AE, Anatskaya OV, 2025; PMID: 41287033, [14]). Nevertheless, here we focus on specific important pathways that are altered in the same direction in both cell types as it allows us to distinguish conserved hallmarks of polyploidy per se from features that are merely contingent on the specific physiological conditions of the cells.

**Figure 6:**
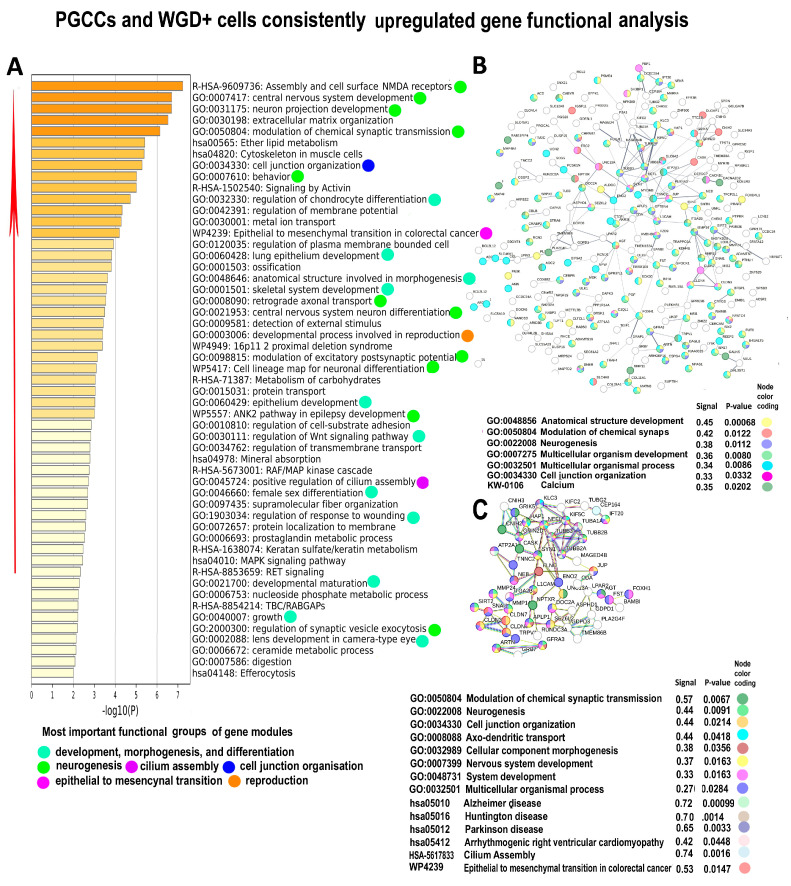
Gene functional enrichment and protein–protein interaction network analysis of genes consistently up-regulated in (PGCCs) and non-giant polyploid cancer cells (WGD+ cells). (**A**) Metascape analysis of genes consistently up-regulated in both PGCCs and non-giant whole-genome-doubled (WGD+) cancer cells. Enriched terms are ranked according to statistical significance (−log10 *p* value). Colored circles indicate the major functional groups of enriched pathways, including development, morphogenesis and differentiation (cyan), neurogenesis (green), epithelial-to-mesenchymal transition (magenta), cilium assembly (purple), cell junction organization (blue), and reproduction (orange). (**B**) Protein–protein interaction network of the consistently up-regulated genes. Nodes represent individual genes, while edges indicate known functional associations. Node colors correspond to the most significantly enriched biological processes or pathways associated with each gene. The inset summarizes the principal enriched functional modules, their enrichment signal, and statistical significance (*p*-value), highlighting developmental processes, neurogenesis, cell junction organization, multicellular organism development, modulation of chemical synaptic transmission, and calcium signaling. (**C**) Subnetwork showing the most highly interconnected functional module identified among the up-regulated genes. This module is enriched for biological processes related to modulation of chemical synaptic transmission, neurogenesis, cell junction organization, axo-dendritic transport, cellular component morphogenesis, and nervous system development, as well as disease-associated pathways including Alzheimer’s disease, Huntington disease, Parkinson disease, arrhythmogenic right ventricular cardiomyopathy, cilium assembly, and epithelial-to-mesenchymal transition. Enrichment signal and corresponding *p*-values are indicated.

**Figure 7:**
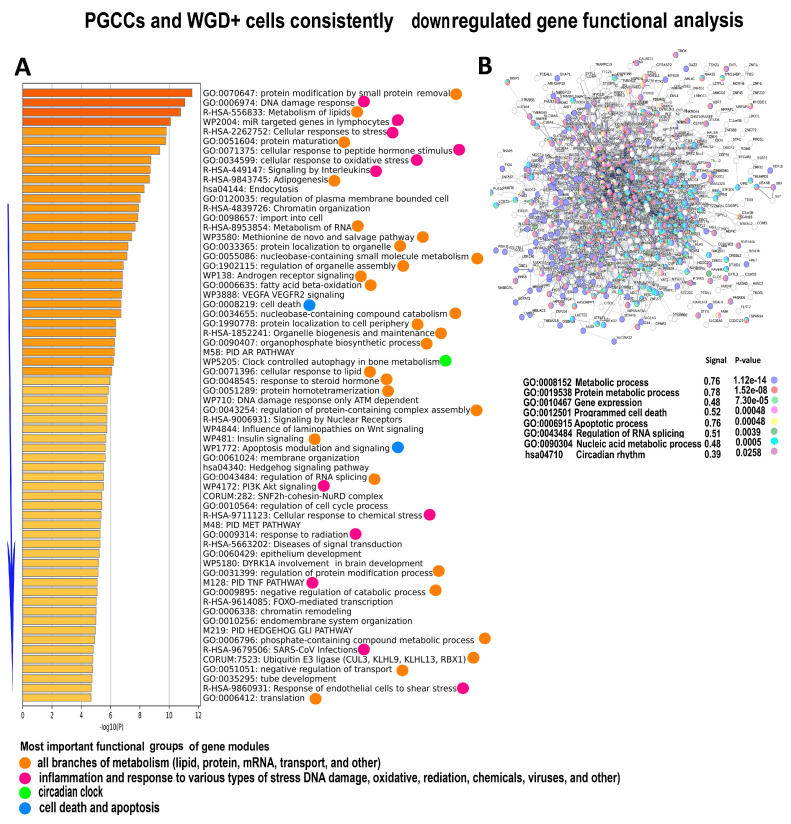
Functional enrichment analysis of genes consistently down-regulated in polyploid giant cancer cells (PGCCs) and non-giant WGD+ cancer cells. (**A**) Functional enrichment analysis of genes consistently down-regulated in both PGCCs and non-giant whole-genome doubled (WGD+) cancer cells using Gene Ontology (GO), Reactome, WikiPathways, KEGG, and PID pathway databases. Enriched biological processes and pathways are ranked according to statistical significance (−log10 *p* value). Colored circles indicate the major functional categories represented by the enriched terms, including metabolic processes (orange), inflammation and cellular responses to diverse stress stimuli—including DNA damage, oxidative stress, radiation, chemical agents, and viral infection (magenta), circadian clock regulation (green), and cell death and apoptosis (blue). (**B**) Functional interaction network of the consistently down-regulated genes. Nodes representing individual genes and edges denote known or predicted functional interactions. Node colors correspond to the predominant enriched biological process or pathway associated with each gene. The inset summarizes the principal functional modules identified within the network, demonstrating significant enrichment for metabolic and protein metabolic processes, gene expression, programmed cell death and apoptosis, regulation of RNA splicing, nucleic acid metabolic processes, and circadian rhythm, together with their corresponding enrichment signal and *p*-values.

To find out whether the obtained PGCCs-related transcriptomic features are conserved and whether they appear in non-giant polyploid cancer cells, we performed the gene-by-gene comparison identifying 243 consistently up-regulated and 681 down-regulated genes shared between the database for PGCCs presented here and the database for WGD+ cancer cells provided by [91]. Supplementary Materials Table S3 and S4 provide consistently up- and down-regulated genes. Metascape analysis of the up-regulated and down-regulated genes showed that both PGCCs and WGD+ cells partially recapitulate the thematic categories previously described in PGCCs, indicating that these transcriptional programs are largely driven by genome doubling itself rather than by cell-type-specific context (Figure 6A–C and Figure 7A,B). For the PGCCs and WGD+ cells’ up-regulated genes, the most striking result is that Metascape identified roughly a dozen gene modules related to neuronality, including neuron differentiation, neuron development, axon guidance, and synaptic transmission (Figure 6). This unexpected convergence suggests that whole-genome doubling (WGD) may broadly activate neuronal-like transcriptional programs outside the nervous system, potentially reflecting a more plastic, less differentiated cellular state. This is consistent with prior work pointing to the reactivation of neural stem cell-like gene expression during cancer cell dedifferentiation [92,93]. Notably, these pro-neuronal pathways converge on a striking cluster of genes encoding synaptic proteins, including DLGAP1, UNC13A, CASK, GRIN2D, GRIK5, APLP1, SCG5, and SPTBN4 (Supplementary Materials Table S3). These proteins are established markers of synaptic identity and maturity [94] rather than upstream drivers of neuronal fate, suggesting that WGD-associated cells acquire the transcriptional signature of differentiated neuronal structures without necessarily being governed by the regulatory programs that specify neuronal lineage. A further pro-neuronal marker is neuron-specific enolase (ENO2), a glycolytic enzyme preferentially expressed in neurons that serves as a metabolic and cytoplasmic indicator of neuronal differentiation and functional maturity [95].

In line with this broader plasticity, genes commonly up-regulated in PGCCs and WGD+ cells were also enriched in pathways related to development, morphogenesis, reproduction, and epithelial-to-mesenchymal transition (Figure 6A). Additional shared modules involved cell junction organization, ciliogenesis, and cilium assembly. STRING analysis of the protein–protein interaction network (PPIN) confirmed the consistency of modules related to neuronality, cell junctions, and development, and further revealed modules associated with multicellularity and calcium signaling (Figure 7B). Because the PPIN contained numerous unconnected genes, we repeated the STRING analysis restricted to genes within the largest connected component of the network (Figure 7B). This analysis reinforced the previously identified modules and additionally uncovered associations with neurodegenerative disorders (Alzheimer’s, Parkinson’s, and Huntington’s diseases), cilium assembly, EMT, cardiomyopathy, and multicellularity.

For the genes down-regulated in PGCCs and WGD+ cells, the most prominent effects uncovered by Metascape reflect features of metabolic deprivation, immunosuppression, and impaired stress response. Metabolic deprivation is evident from the down-regulation of multiple gene modules involved in the metabolism and transport of proteins, lipids, RNA, nucleotides, and amino acids, as well as mRNA splicing (Figure 7A). Features of immunosuppression and impaired stress response are reflected in a group of gene modules governing the response to inflammation and to stress induced by oxidative damage, radiation, chemical agents, and other insults. The Metascape analysis also revealed down-regulation of gene modules related to apoptosis and circadian rhythm (Figure 7A). PPIN analysis further confirmed these functional groups, corroborating the down-regulation of modules related to metabolism, apoptosis, mRNA splicing, and the circadian clock.

Together, these convergent signatures are consistent with genome doubling being associated with a broadly plastic, developmentally primitive, and metabolically quiescent transcriptional state that co-occurs with up-regulation of neuronal-, morphogenetic-, and multicellularity-associated gene modules and down-regulation of metabolic, immune, and apoptotic-checkpoint gene modules, in both PGCCs and WGD+ cells. We propose, as a hypothesis rather than an established mechanism, that this dual signature could contribute to the stem-like, treatment-resistant phenotype characteristic of PGCCs.

## 3. Discussion

The main observation of this study is that PGCCs display a transcriptomic signature in which pro-inflammatory gene modules are co-expressed with gene modules associated, in other contexts, with immune evasion; we interpret this co-expression as consistent with a coordinated transcriptional architecture, though this interpretation extends beyond what the transcriptomic data directly demonstrate. Pro-inflammatory gene modules included cytokine-, interleukin-, and antiviral-signaling genes, and were co-expressed with gene modules associated with cell migration and locomotion, consistent with findings from previous studies [46,55]. We propose that this candidate immune-evasion signature is primarily linked to PD-L1-checkpoint-associated gene expression and an interferon-stimulated/innate immune-response gene signature that pathway databases additionally annotate to virus-response programs. Accumulating evidence in the field is only beginning to shed light on a potential capacity of PGCCs for reduced immune recognition [16,19,96], while the underlying molecular mechanisms remain largely unresolved. This combination of pro-inflammatory phenotype with features of immune evasion resembles the phenotype of highly invasive cancer cells that are cytolytic yet immunologically invisible [40]. This paradoxical phenotype arises when tumor cells acquire active tissue-destructive machinery, such as up-regulated cytolytic gene expression, alongside immune escape mechanisms [1]. Specifically, the cytolytic machinery activates pro-inflammatory cytokine signaling including interleukins, TNF-α, IFN-γ, and interferon-stimulated genes that collectively amplify cytotoxic responses and tissue destruction, while the immunological invisibility is sustained through secretion of immunosuppressive cytokines and, crucially, up-regulation of immune checkpoint pathways mediated by PD-L1 (CD274) expression, disabling both T-cell- and NK-cell-mediated clearance [40]. The phenotypic similarity between PGCCs and cytolytic tumor cells is consistent with, though does not establish, comparably strong cytolytic and immune-evasive capacity; based on this expression signature, we hypothesize that PGCCs could be well equipped for invasion and metastatic spread. Beyond classical immune-regulatory pathways, PGCCs express a broad spectrum of gene modules associated with immune evasion and long-term persistence in other contexts, including dormancy networks, SASP, developmental plasticity, reproductive gene modules, neuronal-associated genes, and apoptosis-resistance-associated genes. Notably, this repertoire maps onto established cancer hallmarks [97], raising the hypothesis that PGCCs could show greater virulence than their diploid counterparts, a possibility not directly tested here. At the same time, although many of these features have been investigated individually [55,98], integrative wide-scale comparative analyses unifying them within a single framework, to our knowledge, are absent. Our data show that these gene modules are co-activated rather than independently activated, and that they share upstream regulatory hubs within the reconstructed network. We interpret this co-activation as consistent with a stress-induced, coordinated transcriptional state in which distinct programs converge, though whether this coordination collectively generates resilience beyond that of any single program is a hypothesis, not a finding directly established by these data.

### 3.1. Senescence-, Developmental-, and Germline-Associated Gene Programs as Candidate Contributors to PGCC Immune-Adaptive Persistence

Within this convergent expression architecture, three co-expressed gene programs, senescence-associated, developmental, and germline/reproductive reprogramming signatures, emerge as candidates that may collectively contribute to PGCC persistence [50]. Based on their coordinated enrichment, we propose a conceptual framework in which these programs could interact, although the present transcriptomic data do not demonstrate functional interactions. In this hypothetical framework, these programs could enable genomically and epigenomically unstable tumor cells not only to survive but also to maintain stable perturbations, consistent with observations reported elsewhere [50]. Each program has been independently associated with mechanisms that may reduce immune recognition. SASP remodels the microenvironment toward immune tolerance [99], developmental programs down-regulate MHC-I and up-regulate PD-L1 [100,101], and germline reprogramming recapitulates the immune privilege of male organs of reproduction [102]. In addition, transcriptomic enrichment of ciliogenesis-related pathways raises the possibility that cilia-associated signaling could contribute to this broader immune-adaptive landscape, as primary cilia integrate embryonality-related signals and have been implicated in immune regulation [103]. Because these programs are co-enriched, it is tempting to speculate that they may cooperate rather than operate independently. However, such cooperation cannot be inferred from co-expression alone. SASP-derived cytokines acting through interleukins, STATs, and NF-κB can activate embryonality and germline reprogramming, thereby preventing senescence from becoming terminal [104,105]. We propose, as a testable model rather than an established mechanism, that these interactions could constitute a self-stabilizing, three-node immunosuppressive circuit in which each program reinforces the others, potentially producing a layered immune-evasive state that is more robust than would be expected from the individual programs alone. At present, however, this putative circuit is supported by coordinated transcriptional signatures rather than direct evidence of functional interdependence or causality.

This model currently rests on co-enrichment and gene-module analyses. Therefore, establishing causality will require two complementary functional approaches. The first is single-node perturbation or knockdown of core developmental regulators in PGCC-enriched populations, with MHC-I/PD-L1 expression measured by flow cytometry. This approach can verify whether each program is individually required to drive the immune-evasive phenotype, as presented in studies of developmental/EMT transcription factors [106]. The second approach is in vivo validation in syngeneic or humanized xenograft models, comparing single- versus combinatorial-node knockdowns for effects on CD8+ T-cell infiltration, checkpoint expression, and tumor growth. This approach can help to understand whether the circuit’s synergy holds in a physiological setting and is required for its full immune-evasive effect, consistent with syngeneic models in which single-gene knockdown alone was sufficient to enhance CD8+ T-cell infiltration and suppress tumor growth [107]. Together, these loss-of-function studies would move the three-node model from a correlative framework to a testable mechanistic hypothesis, clarifying whether its nodes act redundantly or must be disrupted simultaneously to overcome their reported synergy.

### 3.2. Dormancy as a Program Promoting Immune Escape

The programs of inflammation, immune evasion, senescence, development, and germline are closely associated with transcriptional programs that have previously been linked to another hallmark of PGCC biology, namely, dormancy-associated states. Tumor dormancy has been reported to be associated with immune evasion, with dormant cells in some experimental systems exhibiting checkpoint activation, MHC-I down-regulation, and escape from NK-cell surveillance [108,109]. In the present study, however, we identify transcriptional signatures consistent with dormancy-associated biology rather than directly demonstrating a dormant cellular state. Specifically, our transcriptomic analysis revealed coordinated down-regulation of gene modules involved in protein synthesis, metabolism, RNA processing, and cell-cycle progression. These changes are compatible with previously described dormancy-associated transcriptional programs but do not, by themselves, establish functional dormancy, as no cell-cycle analyses, proliferation assays, live-cell imaging, or lineage-tracing experiments were performed. Accordingly, these findings should be interpreted as evidence of dormancy-associated transcriptional signatures rather than confirmed dormancy. The observed transcriptional profile also shares similarities with gene-expression programs reported during embryonic diapause described by Liu and colleagues [110], a reversible low-metabolic state associated with preserved developmental potential. Although this resemblance is intriguing, the present data do not establish that PGCCs undergo an analogous biological state.

If these dormancy-associated transcriptional programs correspond to bona fide dormant states, they could provide a mechanism by which PGCCs retain the capacity for subsequent reactivation in response to favorable environmental conditions. This possibility remains to be tested experimentally using functional approaches. Likewise, the enrichment of anti-apoptotic pathways identified in our analysis should be interpreted as evidence of apoptosis-resistance-associated transcriptional signatures rather than direct demonstration of apoptosis resistance.

Taken together, the coordinated enrichment of dormancy- and apoptosis-resistance-associated transcriptional signatures suggests that PGCCs may possess molecular features compatible with long-term persistence. However, whether these signatures translate into functional dormancy, enhanced survival, or relapse capacity will require direct experimental validation. Given that metastatic relapse may occur years or even decades after apparent clinical remission, these transcriptional programs provide a conceptual framework for investigating mechanisms that may contribute to persistent cancer cell reservoirs and late disease recurrence.

### 3.3. Apoptosis Resistance Decreases Immune Recognition

Our results also suggest that PGCCs exhibit a transcriptomic profile consistent with reduced apoptotic susceptibility rather than providing direct evidence of apoptosis resistance. Specifically, PGCCs are enriched for anti-apoptotic transcriptional signatures, while transcriptional programs associated with pro-apoptotic pathways appear suppressed. Experimental evidence from previous studies has shown that PGCCs induced by chemotherapy or radiation can exhibit reduced sensitivity to classical apoptosis despite extensive cell damage [81,111,112]. Although our transcriptomic analysis does not directly assess apoptotic susceptibility, the observed expression patterns are consistent with these published functional observations. This phenotype can protect PGCCs from immune surveillance [81] and, together with our transcriptomic findings, we speculate that it may reflect a broader strategy in which damage tolerance and survival take precedence over genomic integrity, potentially reinforcing the resilience of these cells [14].

### 3.4. Neuronal- and Neurodegeneration-Associated Gene Programs as Candidate Contributors to Immune Evasion

An unexpected expansion of the PGCC-associated adaptive landscape is the activation of signaling programs annotated in neuronal development, synaptic regulation, and neural-associated processes. Increasing evidence shows that cancer cells can co-opt neural communication systems (“cancer-neuron mimicry”) to enhance fitness and modulate tumor–immune interactions [86,111]. However, many genes involved in these pathways, including semaphorins, plexins, developmental regulators, and calcium-signaling molecules, have broad functions beyond the nervous system. Therefore, enrichment of neuronal-associated pathways should not be interpreted as evidence of genuine neuronal reprogramming, but rather as the presence of molecular programs that overlap with neural regulatory networks. Beyond neural development per se, these pathways regulate immune-cell recruitment, spatial organization, and microenvironmental structure [112]. Consistent with this paradigm, PGCCs exhibit coordinated activation of neuronal-associated signaling components, calcium-dependent pathways, and axon-guidance-related networks which have also been reported in independent PGCC studies [63,113]. These observations suggest that PGCCs may utilize evolutionarily conserved signaling modules that are shared between neural development and tumor adaptation. We hypothesize that these systems could link cellular plasticity with spatial control of immune interactions, raising the possibility that neuronal-associated gene expression contributes to immune escape, though this was not tested functionally. From a systems-level perspective, the neuronal signaling repertoire of PGCCs may contribute to regulatory network plasticity, analogous to synaptic plasticity in learning neural networks [114]. This analogy represents a conceptual framework rather than evidence of neuronal-like information processing in PGCCs. Just as connection remodeling underlies learning in both biological and artificial neural networks, the ability of PGCCs to remodel their regulatory interactions may represent a mechanism supporting phenotypic plasticity under selective pressure, although the involvement of neuronal signaling machinery in this process remains speculative. Closely linked to these observations is the enrichment of pathways annotated in neurodegenerative disorders. However, these findings should not be interpreted as indicating that PGCCs acquire a neurodegenerative phenotype. Bioinformatic analyses consistently revealed enrichment of neuronal cytoskeletal components, calcium-dependent signaling, chronic inflammatory pathways, and regulators of neural development that are well-established hallmarks of neurodegeneration [87,88,89]. Because these pathways are also involved in fundamental cellular processes such as cytoskeletal organization, stress responses, calcium homeostasis, and inflammation, their enrichment may reflect broader adaptive mechanisms rather than neurodegeneration per se. Elevated expression of neuronal structural genes may contribute to cytoskeletal adaptation and mechanical stability in the unusually large and structurally complex PGCC architecture. In parallel, persistent inflammatory signaling shares molecular features with chronic inflammatory states described in neurodegenerative disorders, but these similarities do not establish a neurodegenerative process in PGCCs [89]. Importantly, these same inflammatory circuits are well known to promote immunosuppression in tumors [1,109], raising the possibility that this reprogramming overlapping stress- and inflammation-associated programs contributes to tumor adaptation.

Thus, rather than representing a state reminiscent of neurodegenerative processes, PGCCs display enrichment of neuronal- and neurodegeneration-associated molecular signatures that may reflect the repurposing of conserved developmental, cytoskeletal, calcium-regulatory, and inflammatory pathways. We propose this as a speculative model in which such programs could enhance structural resilience and influence immune interactions; however, direct functional studies will be required to determine whether these pathways contribute to immune evasion or other adaptive properties of PGCCs.

### 3.5. Polyploidy-Associated Phenotypes Are Conserved Across Diverse Cell Types

It is important to note that several of the observed PGCC features including reduced immune surveillance, senescence, enhanced migration and EMT, ciliogenesis, apoptosis resistance, programs of development and even immune evasion are not unique to the population of PGCCs but are shared across diverse polyploid cell types. The comparative analysis of the database for PGCCs presented here and the database obtained with cancer WGD+ cells by Quinton, et al., 2021 [91], are consistent with whole-genome doubling (WGD) contributing to the PGCC phenotype rather than being merely a passive by-product of it, although causality cannot be established from cross-sectional transcriptomic comparison. The convergence of up-regulated neuronal, morphogenetic, and multicellularity programs with down-regulated metabolic, immune, and apoptotic modules points to a coordinated shift toward a plastic, developmentally primitive state, i.e., the one that echoes neural stem cell-like dedifferentiation reported in other cancer contexts [92,93]. It is important to note that all of these features, separately or in partial concert have been reported in other types of polyploid cells. Thus, features of EMT, apoptosis resistance, programs of development, and decreased recognition by immunity were recently found in mesenchymal stem cells, cardiac progenitors, cardiomyocytes, hepatocytes, as well as trophoblast giant cells and megakaryocytes [35,115,116,117,118]. Moreover, our recent study performed with adult cardiomyocytes that acquired hyperpolyploidy after neonatal systemic inflammation, revealed the long-term overexpression of CD274 [115] (Supplementary Materials Table S1, cluster of immunity, line 135) a pattern consistent with, and supporting the hypothesis of, an association between polyploidy and immune evasion. Accordingly, a recent study indicated that WGD+ cells trigger immune evasion by silencing antigen presentation via metabolic and epigenetic alterations [119]. The recurrence of common features across contexts such as tumor progression, differentiation, regeneration, and proliferation points to a conserved polyploidy-associated program rather than context-specific adaptations [44,120]. A unifying driver of this program may be DNA instability arising early during polyploidization, particularly at the tetraploid stage [91,121]. Accumulating evidence indicates that DNA damage can induce PD-L1 expression, thereby promoting immune escape [122,123,124]. In parallel, genome instability has been linked to activation of EMT, pro-inflammatory signaling, and programs of development, collectively driving aggressive, therapy-resistant phenotypes [96,125].

### 3.6. Regulatory Plasticity as a Possible Driver of Immune Evasion in PGCCs

The suggestion that DNA instability may contribute to decreased recognition by immunity in PGCCs is consistent with the concept of “genome chaos”, which proposes that under severe stress, large-scale genome reorganization generates heterogeneous karyotypic states that may facilitate rapid adaptation [6]. Within this conceptual framework, the adaptation is thought to involve regulatory plasticity enabling adaptive exploration of gene regulatory network (GRN) states through self-organization [7,11]. Accordingly, genome-level and GRN-level processes may be interconnected, with genome chaos potentially expanding the landscape of accessible regulatory states and favoring the emergence of stable, stress-adaptive attractors such as the PGCC phenotype [126,127,128,129,130]. The presence of multiple nuclei and extensive genomic instability in PGCCs may further increase regulatory heterogeneity, potentially facilitating the emergence of treatment-resistant transcriptional states. However, these mechanisms remain hypothetical and were not directly examined in the present study.

Consistent with this framework, persistent genomic instability in PGCCs may simultaneously sustain inflammatory signaling while promoting checkpoint-mediated immune suppression. This coupling could provide a plausible mechanistic bridge between stress activation, genome restructuring, and reduced immune clearance, consistent with the hypothesis that PGCCs represent a state in which gene modules associated with inflammation and immune evasion are coordinately regulated, a hypothesis that remains to be functionally tested.

### 3.7. Clinical Applications

If the proposed coordinated regulatory architecture of PGCCs is validated functionally, the most promising therapeutic strategy would likely be to target shared upstream hubs that govern multiple arms of the candidate immune-evasion network. NF-κB, STAT3, and cytokines are prime candidates, serving as central regulators of SASP, PD-L1 transcription, developmental reprogramming, pro-inflammatory signaling, dormancy, and apoptosis resistance. Their inhibition could therefore collapse multiple, reinforcing layers of immune evasion simultaneously. HIF1A, which integrates hypoxic stress with checkpoint activation and dormancy enforcement, represents another high-leverage node. Calcium signaling, sitting at the intersection of neuronal mimicry, transcriptional plasticity, and immune suppression, offers further network-level vulnerability accessible with clinically available agents. This convergence is clinically significant: anti-neurodegenerative drugs, many of which were developed to modulate calcium flux and stabilize aberrant neuronal signaling, hold considerable and largely untapped medical potential for repurposing in PGCC-targeted therapy, given the striking overlap between the calcium-dependent survival circuitry of PGCCs and the pathways these agents were originally designed to modulate. Targeting these shared hubs, rather than individual pathways, would simultaneously disrupt the putative feedback-stabilized regulatory state, restore immune visibility, and block the reactivation of dormant PGCCs that drives relapse.

Because these regulatory modules are highly interconnected, inhibition of a single pathway is likely to trigger compensatory activation of parallel inflammatory, developmental, or metabolic programs that preserve PGCC survival. Consequently, rational combination strategies should target a central regulatory hub together with its most probable adaptive escape mechanisms. For example, inhibition of NF-κB/STAT3 signaling may be combined with immune checkpoint blockade or agents targeting calcium-dependent plasticity to prevent re-establishment of immune-evasive or dormant states. A logical therapeutic sequence would first weaken the stress-adaptive regulatory network and subsequently enhance immune-mediated tumor elimination, with treatment timing guided by biomarkers reflecting inflammatory activity, checkpoint expression, and adaptive pathway engagement.

### 3.8. Limitations of the Study

We should acknowledge that our study has several limitations. First, our findings are derived entirely from a transcriptomic meta-analysis, which captures gene expression changes but does not directly measure protein abundance or post-translational modification. Because transcript and protein abundance are often only moderately correlated, changes in mRNA expression do not necessarily translate into corresponding changes in protein levels or biological activity. Moreover, many signaling pathways are regulated through post-translational mechanisms, particularly protein phosphorylation, which cannot be inferred from transcriptomic data alone. Consequently, the present study lacks both proteomic and phosphoproteomic validation of the predicted molecular networks. As such, the biological pathways and regulatory relationships proposed here should be considered correlative and hypothesis-generating rather than mechanistically established. Second, transcriptomic data alone cannot determine whether observed expression changes are a cause, consequence, or bystander effect of the phenotype under investigation. Functional perturbation studies are needed to establish causality. Third, although we applied a recurrence-based meta-analysis across multiple independent datasets to identify robust and conserved transcriptional signatures, the included studies differ in cancer type, experimental design, sequencing platform, sample preparation, and analytical pipelines. In addition, methods used to generate and isolate PGCCs are not fully standardized across studies, introducing potential biological and technical heterogeneity that may influence the detected transcriptional profiles. Fourth, pathway-enrichment analyses are inherently dependent on existing knowledge bases and curated annotation databases. Because many genes participate in multiple biological processes, enriched pathways should not be interpreted as direct evidence for activation of specific cellular programs. In particular, annotations related to neuronal development, synaptic signaling, neurodegeneration, viral infection, and SARS-CoV-2 pathways frequently share genes involved in broader processes such as calcium signaling, stress adaptation, interferon responses, inflammation, and developmental regulation. Therefore, these enrichments should be interpreted as evidence of shared transcriptional modules rather than definitive proof of neuronal reprogramming, reminiscent of neurodegenerative processes, viral mimicry, or virus-specific biological processes. Finally, the present study was designed to identify conserved transcriptional architectures rather than to establish functional mechanisms. The molecular programs proposed here should therefore be regarded as experimentally testable hypotheses that require validation using complementary approaches, including proteomics, phosphoproteomics, functional perturbation studies, single-cell and spatial transcriptomics, and in vitro and in vivo experimental models.

### 3.9. Perspectives

Translating the presented network-level landscape into mechanism will require following the data from transcript to protein to cell behavior. Co-immunoprecipitation can put the model’s central wiring to the test, asking whether GSK3B and PD-L1 physically engage as predicted and whether NF-κB and STAT3 truly assemble into the hub complex our networks imply. Phosphoproteomics adds the missing dimension of activity. Since a transcript’s presence says nothing about whether its protein is switched on, mapping the phosphorylation states of STAT3, NF-κB, and HIF1A would reveal whether these nodes are merely co-expressed or actively signaling in concert. CRISPR perturbation screens would then let us interrogate the circuit’s logic directly including silencing each hub regulator in turn and watching for shifts in MHC-I/PD-L1 expression and vulnerability to CD8+ T-cell killing to determine whether these nodes act as redundant safety nets or as indispensable linchpins the tumor cannot survive without. Finally, because a neuronal gene signature is only a hypothesis until cells are watched in real time, live-cell calcium imaging would show whether PGCCs actually behave like the excitable, calcium-responsive cells their transcriptomes suggest, closing the loop between a computational prediction and a living phenotype.

## 4. Materials and Methods

### 4.1. Datasets

We analyzed transcriptomes of PGCCs vs. diploid cancer cells lines derived from prostate, ovarian, and breast cancer tissues. The datasets present different diploid cancer cell lines where PGCCs were obtained by various treatments. The datasets were the same as in our previous study where the analysis was performed mostly on the level of multi-gene signatures [14]. Here, we performed in-depth by-gene analysis of consensus (consistently changed in most datasets) genes in the PGCC vs. parent diploid cancer cells. The transcriptome databases of polyploid giant cancer cells (PGCC) acquired from the Gene Expression Omnibus (GEO) [131] were characterized in our previous work [14]. Here, we describe them briefly. In total, there are eight datasets derived from four databases (based on four published papers). They are characterized in Supplementary Materials Table S5. The first database was acquired from the work [55] (GEO accession GSE178745). In that study, the two PGCCs were generated from two established high-grade serous ovarian cancer cell lines, HEY and SKOV3, following paclitaxel treatment. The second database originated from the work [43] (GEO accession GSE229119). Here, PGCCs were induced by olaparib, a poly(ADP-ribose) polymerase (PARP) inhibitor, across a range of model systems: established ovarian and breast cancer cell lines as well as primary cells from individual high-grade serous ovarian cancers maintained in patient-derived xenografts. For the present study, we used transcriptomic data from PGCCs derived from organoid 2414 (Org-2414) (thus giving three datasets). The third database came from the work [113] (GEO accession GSE196453) where PGCCs were induced in prostate cell line by radiation. The fourth database was obtained from the study [132] (GEO accession GSE195919). Here, PGCCs were produced by exposure of parental cells to a single 8 Gy dose of gamma irradiation. In parallel experiments, the cells were also treated with LCL521 that is a pro-drug that inhibits the lysosomal enzyme acid ceramidase (ASAH1). This gives two datasets from this database. In total, there are eight datasets. For consistency, we used the first time point (3 d) after treatment for all compared datasets. For better comparability across datasets, the data were log-transformed and standardized, i.e., expressed in standard deviations with mean as zero (thus removing possible differences in means and variances), as previously [14,15]. To perform transcriptome comparison between PGCCs and non-giant polyploid cancer cells, we retrieved the data from the database (one integrative pan-cancer dataset) provided by Quinton, et al., 2021 [91].

### 4.2. Cross-Study Meta-Analysis for Consistent DEG Identification

Recognizing the substantial heterogeneity inherent to cancer transcriptomes, we implemented a multi-dataset integrative strategy designed to identify robust and conserved PGCC-associated transcriptional programs. To maximize robustness and reproducibility, we focused on genes consistently over- or under-expressed across the majority of datasets. We chose this approach because reproducibility across diverse populations of PGCCs and experimental platforms filters out noise and study-specific confounders, leaving only genes whose deregulation reflects a fundamental, stable aspect of the underlying disease biology. This approach is widely used in cross-study transcriptomic meta-analysis to prioritize reproducible signals over dataset-specific effects [133,134]. The main benefits of this approach are as follows: (1) Dataset heterogeneity is neutralized, not merely accounted for. Vote-based consensus is fundamentally robust to individual dataset idiosyncrasies: a gene only survives if it is independently recovered across multiple methods/datasets [135]. This is a structurally stronger safeguard than statistical adjustment for heterogeneity, which can only correct for known sources of variance. Our approach is immune to both known and unknown sources. (2) Domination by any single dataset is mathematically precluded. Because inclusion requires majority agreement, no single study regardless of size, effect magnitude, or noise can unilaterally drive the signature. This is a stronger and more direct guarantee of robustness than any single-dataset sensitivity analysis could demonstrate, since it prevents the failure mode outright rather than detecting it after the fact [136]. (3) Reproducibility is the defining property of the method, not an incidental feature. Effect-size and random-effects meta-analytic models optimize for statistical power, often at the cost of amplifying signals from outlier or high-variance studies. Our approach optimizes directly for cross-study reproducibility, which is the more clinically and biologically meaningful criterion when the goal is identifying dependable transcriptional signatures. Enrichment stability is a direct, provable consequence. Since enrichment analyses operate downstream of an already-validated, high-confidence gene set (from each database, we filtered out genes with FDR < 0.05), instability at the enrichment level would only reflect instability that had already survived our consensus filter, a considerably higher bar than enrichment performed on unfiltered or single-method DEG lists. Here, we applied transcriptomic meta-analysis combining *p*-value integration and vote-counting approaches. Specifically, according to the Fisher’s combined *p*-value principle, we aggregated gene *p*-values across datasets [137]. Genes were classified as consistently up- or down-regulated if they were expressed in at least five datasets (from eight), showed a concordant direction of change (up or down) across the majority of datasets where they were expressed (≥5 dataset concordance), and their upward or downward expression change met the significance threshold of false discovery rate (FDR), The integrated FDRs for up- or down-regulated genes turned out to be very high (FDR < 0.10^−10^). They were calculated on the ground of multiplied primary *p*-values for up- or down-regulated genes separately. Notably, for genes with cross-dataset concordance below 100%, the combined significance (log10 FDR) was above a dozen orders of magnitude higher for the accepted than for the opposite direction (15.4 ± 1.5 for up-regulation vs. down-regulation; 23.2 ± 1.1 for down-regulation vs. up-regulation). Genes showing discordant regulation were excluded from the core consistent signature. Stringent concordance criteria including expression in at least five datasets and unidirectional agreement in at least five datasets ensures that only robustly reproducible signals were retained, effectively filtering out dataset-specific variation (including differences in tissues, cell lines, and treatments).

### 4.3. Protein–Protein Interaction Network

The consistently deregulated gene sets were mapped onto the STRING protein–protein interaction (PPI) database (version 12.0; https://string-db.org/, assessed on 16 April 2026) using a high interaction confidence score of 0.7. To identify biologically central regulators, we applied degree-based hub gene identification within protein–protein interaction networks. Genes falling within the top 20% of degree distribution were classified as hub genes, following established recommendations [138,139,140]. This strategy was selected because highly connected nodes in biological networks are more likely to represent essential or functionally central components governing cellular processes [141,142]. These top up- and down-regulated hub genes were selected for further analysis. The up-regulated PGCC hub genes (*n* = 199) are presented in Supplementary Materials Table S1, the down-regulated PGCC hub genes (*n* = 240) are shown in Supplementary Materials Table S2. Molecular Complex Detection (MCODE) was applied using Metascape [143] to identify densely connected protein complexes within the PPI network, using default parameters (degree cutoff = 2, node score cutoff = 0.2, K-core = 2, maximum depth = 100). Network visualization and topology analysis were performed using STRING and Cytoscape (version 3.10).

### 4.4. Functional Enrichment Analysis

Functional enrichment analysis of consistently up-regulated and down-regulated gene sets was performed using Metascape (v3.5.20260201) and STRING (Version 12.0) where Gene Ontology Biological Process, KEGG, Reactome, and WikiPathways databases were used as annotation sources. Enrichment was considered significant at *p* < 0.01 after Benjamini–Hochberg correction, with a minimum overlap of three genes per term. To reduce redundancy, enriched terms were clustered by semantic similarity, and representative terms were selected for visualization. The results of functional enrichment analysis performed with the STRING server were sorted by the ‘signal’ parameter, which was recently developed by the STRING team for better selecting the most important results [144]. The ‘signal’ is defined as a weighted harmonic mean between −log10 FDR and the ‘strength’. The FDR is the false discovery rate, obtained by the Benjamini–Hochberg procedure. The ‘strength’ is the log10 observed/expected gene number. This was necessary because FDR tends to emphasize larger enriched gene groups due to their potential for achieving lower *p*-values, while the observed/expected ratio highlights smaller groups, which have a high foreground to background ratio but cannot achieve low FDR values due to the small group size. The ‘signal’ measure seeks to balance both metrics for more meaningful ordering of enriched groups. The analyses and visualization were performed using Statgraphics Centurion, R package, Cytoscape, Metascape, and STRING server.

## 5. Conclusions

This meta-analysis reveals a conserved gene-expression signature across independent PGCC datasets, in which pro-inflammatory, immune-evasion, developmental, and neuronal gene modules are co-expressed and linked through shared regulatory hubs. We propose, as a testable hypothesis rather than an established mechanism, that PGCCs organize these modules into a coordinated, multilayered transcriptional architecture supporting survival under therapeutic stress. Co-expression patterns point to several candidate mechanistic links warranting direct functional testing. Stress response and genomic instability correlate with interferon-stimulated gene expression and IRF/STAT-associated PD-L1 expression, while GSK3B down-regulation co-occurs with signatures consistent with reduced PD-L1 turnover—together suggesting a possible route by which inflammatory signaling could couple to durable immune escape. Building on a proposed three-node senescence–developmental–germline model, we find that genes from each program co-express with immune-regulatory genes. Whether each node independently suppresses immune recognition, and whether their combination yields immunosuppressive capacity beyond any single component, remains to be tested experimentally. A neuronal and neurodegeneration-associated signature offers a further candidate layer: semaphorin-plexin expression is consistent with a role in spatially restricting immune infiltration, and calcium-signaling gene expression is consistent with enhanced plasticity and stress resilience—though neither role was directly tested here. PGCC dormancy-associated expression represents an additional candidate layer that may link reduced immune visibility to long-term relapse risk. At the systems level, we hypothesize that genome chaos acts as a unifying driver of this architecture, with stress-induced genome reorganization expanding the accessible regulatory landscape and stabilizing PGCCs in a stress-adaptive state where inflammatory and immunosuppressive programs are coordinately expressed. Together, these findings define a conserved, testable framework—not a demonstrated mechanism. The functional and causal experiments outlined in Section 3.8 and Section 3.9 are the necessary next steps to determine whether, and how, these co-expressed programs drive immune evasion and PGCC persistence.

## Data Availability

The original contributions presented in this study are included in the article/Supplementary Materials. Further inquiries can be directed to the corresponding authors.

## Supplementary Materials

The following supporting information can be downloaded at: https://www.mdpi.com/article/10.3390/ijms27156671/s1.

## References

[1] Galassi C., Chan T.A., Vitale I., Galluzzi L. (2024). The Hallmarks of Cancer Immune Evasion. Cancer Cell.

[2] Tufail M., Jiang C.-H., Li N. (2025). Immune Evasion in Cancer: Mechanisms and Cutting-Edge Therapeutic Approaches. Signal Transduct. Target. Ther..

[3] Greten F.R., Grivennikov S.I. (2019). Inflammation and Cancer: Triggers, Mechanisms, and Consequences. Immunity.

[4] Zhao H., Wu L., Yan G., Chen Y., Zhou M., Wu Y., Li Y. (2021). Inflammation and Tumor Progression: Signaling Pathways and Targeted Intervention. Signal Transduct. Target. Ther..

[5] Gonye A.L., Orzolek L., Cherry C., Patatanian M., Loftus L.V., Truskowski K., Butler G., Pienta K.J., Amend S.R. (2026). Polyploid Cisplatin-Resistant Cancer Cells Have Altered Nuclear Organization and Epigenomic Status. Neoplasia.

[6] Horne S.D., Ye J.C., Liu G., Abdallah B.Y., Stevens J.B., Heng H.H. (2026). Therapy-Induced Rapid Drug Resistance Driven by Genome Chaos and Polyploid Giant Cancer Cells. Cancer Lett..

[7] Pisco A.O., Huang S. (2015). Non-Genetic Cancer Cell Plasticity and Therapy-Induced Stemness in Tumour Relapse: “What Does Not Kill Me Strengthens Me”. Br. J. Cancer.

[8] Tsuchiya M., Giuliani A., Yoshikawa K. (2020). Cell-Fate Determination from Embryo to Cancer Development: Genomic Mechanism Elucidated. Int. J. Mol. Sci..

[9] Vainshelbaum N.M., Giuliani A., Salmina K., Pjanova D., Erenpreisa J. (2022). The Transcriptome and Proteome Networks of Malignant Tumours Reveal Atavistic Attractors of Polyploidy-Related Asexual Reproduction. Int. J. Mol. Sci..

[10] Erenpreisa J., Salmina K., Vainshelbaum N.M., Inashkina I., Freivalds T. (2024). Self-Organisation of Early Stress Response in the Biology of Cancer. Postep. Biochem..

[11] Erenpreisa J., Salmina K., Anatskaya O., Cragg M.S. (2022). Paradoxes of Cancer: Survival at the Brink. Semin. Cancer Biol..

[12] Tsuchiya M., Brazhnik P., Bizzarri M., Giuliani A. (2023). Synchronization between Attractors: Genomic Mechanism of Cell-Fate Change. Int. J. Mol. Sci..

[13] Carvalho J. (2025). Evidence for the Atavistic Reversal to a Unicellular-like State as a Central Hallmark of Cancer. Int. J. Cancer.

[14] Vinogradov A.E., Anatskaya O.V. (2025). Polyploid Giant Cancer Cells (PGCC): Short-Term Return to Multicellularity. Biol. Res..

[15] Vinogradov A.E., Anatskaya O.V. (2025). “Cell Dedifferentiation” versus “Evolutionary Reversal” Theories of Cancer: The Direct Contest of Transcriptomic Features. Int. J. Cancer.

[16] Liu J., Liu K., Yan M., Xu J., Liu Y., Zhang S. (2026). Dormant and Activated Polyploid Giant Cancer Cells: From Chemoradiotherapy Resistance to Cancer Progression. Cancer Lett..

[17] Mirzayans R., Murray D. (2022). What Are the Reasons for Continuing Failures in Cancer Therapy? Are Misleading/Inappropriate Preclinical Assays to Be Blamed? Might Some Modern Therapies Cause More Harm than Benefit?. Int. J. Mol. Sci..

[18] Ogawa Y., Fisher L., Matsumoto T. (2025). The Impact of Polyploid Giant Cancer Cells: The Root of Stress Resilience. Cancer Sci..

[19] Chen J., Niu N., Zhang J., Qi L., Shen W., Donkena K.V., Feng Z., Liu J. (2019). Polyploid Giant Cancer Cells (PGCCs): The Evil Roots of Cancer. Curr. Cancer Drug Targets.

[20] Zhang S., Mercado-Uribe I., Xing Z., Sun B., Kuang J., Liu J. (2014). Generation of Cancer Stem-like Cells through the Formation of Polyploid Giant Cancer Cells. Oncogene.

[21] Niu N., Mercado-Uribe I., Liu J. (2017). Dedifferentiation into Blastomere-like Cancer Stem Cells via Formation of Polyploid Giant Cancer Cells. Oncogene.

[22] Zhang S., Mercado-Uribe I., Hanash S., Liu J. (2013). iTRAQ-Based Proteomic Analysis of Polyploid Giant Cancer Cells and Budding Progeny Cells Reveals Several Distinct Pathways for Ovarian Cancer Development. PLoS ONE.

[23] Moein S., Adibi R., da Silva Meirelles L., Nardi N.B., Gheisari Y. (2020). Cancer Regeneration: Polyploid Cells Are the Key Drivers of Tumor Progression. Biochim. Biophys. Acta Rev. Cancer.

[24] Schmidt M.J., Naghdloo A., Prabakar R.K., Kamal M., Cadaneanu R., Garraway I.P., Lewis M., Aparicio A., Zurita-Saavedra A., Corn P. (2025). Polyploid Cancer Cells Reveal Signatures of Chemotherapy Resistance. Oncogene.

[25] Wang G., Wang H., Wang Y., Li X., Luo H. (2026). Polyploid Giant Cancer Cells: A Novel Target in Future Cancer Therapy. Eur. J. Cell Biol..

[26] Zhao Y., Lu T., Song Y., Wen Y., Deng Z., Fan J., Zhao M., Zhao R., Luo Y., Xie J. (2023). Cancer Cells Enter an Adaptive Persistence to Survive Radiotherapy and Repopulate Tumor. Adv. Sci..

[27] Trigos A.S., Bongiovanni F., Zhang Y., Zethoven M., Tothill R., Pearson R., Papenfuss A.T., Goode D.L. (2024). Disruption of Metazoan Gene Regulatory Networks in Cancer Alters the Balance of Co-Expression between Genes of Unicellular and Multicellular Origins. Genome Biol..

[28] Trigos A.S., Pearson R.B., Papenfuss A.T., Goode D.L. (2017). Altered Interactions between Unicellular and Multicellular Genes Drive Hallmarks of Transformation in a Diverse Range of Solid Tumors. Proc. Natl. Acad. Sci. USA.

[29] Vinogradov A.E., Anatskaya O.V. (2023). Systemic Alterations of Cancer Cells and Their Boost by Polyploidization: Unicellular Attractor (UCA) Model. Int. J. Mol. Sci..

[30] Ichiryu N., Fairchild P.J. (2013). Immune Privilege of Stem Cells. Methods Mol. Biol..

[31] Mullins R.D.Z., Pal A., Barrett T.F., Heft Neal M.E., Puram S.V. (2022). Epithelial-Mesenchymal Plasticity in Tumor Immune Evasion. Cancer Res..

[32] Porcelli G., D’Accardo C., Angeloro F., Cucchiara M., Bianca P., Pantina V.D., Roozafzay N., Modica C., Gaggianesi M., Di Bella S. (2025). Decoding Cancer Dormancy: Integrative Genomic, Phenotypic and Live-Cell Imaging Analysis to Reveal the Hidden Cancer Cell Reservoir. Mol. Cancer.

[33] Niculescu V.F. (2023). Cancer Exploits a Late Premetazoan Gene Module Conserved in the Human Genome. Genes. Dis..

[34] Trigos A.S., Pearson R.B., Papenfuss A.T., Goode D.L. (2019). Somatic Mutations in Early Metazoan Genes Disrupt Regulatory Links between Unicellular and Multicellular Genes in Cancer. eLife.

[35] Anatskaya O.V., Ponomartsev S.V., Elmuratov A.U., Vinogradov A.E. (2025). Transcriptome-Wide Insights: Neonatal Lactose Intolerance Promotes Telomere Damage, Senescence, and Cardiomyopathy in Adult Rat Heart. Int. J. Mol. Sci..

[36] Anatskaya O.V., Vinogradov A.E. (2022). Polyploidy as a Fundamental Phenomenon in Evolution, Development, Adaptation and Diseases. Int. J. Mol. Sci..

[37] Pienta K.J., Hammarlund E.U., Austin R.H., Axelrod R., Brown J.S., Amend S.R. (2022). Cancer Cells Employ an Evolutionarily Conserved Polyploidization Program to Resist Therapy. Semin. Cancer Biol..

[38] Li X., Zhong Y., Zhang X., Sood A.K., Liu J. (2023). Spatiotemporal View of Malignant Histogenesis and Macroevolution via Formation of Polyploid Giant Cancer Cells. Oncogene.

[39] Manickasamy M.K., Aswani B.S., Banerjee R., Abbas M., Alqahtani M.S., Sethi G., Liu L., Kunnumakkara A.B. (2026). Etiology of Polyploid Giant Cancer Cells: A New Frontier in Cancer Biology. Cancer Cell Int..

[40] Rooney M.S., Shukla S.A., Wu C.J., Getz G., Hacohen N. (2015). Molecular and Genetic Properties of Tumors Associated with Local Immune Cytolytic Activity. Cell.

[41] Haidar Ahmad S., El Baba R., Herbein G. (2023). Polyploid Giant Cancer Cells, Cytokines and Cytomegalovirus in Breast Cancer Progression. Cancer Cell Int..

[42] Kudo-Saito C., Miyamoto T., Imazeki H., Shoji H., Aoki K., Boku N. (2020). IL33 Is a Key Driver of Treatment Resistance of Cancer. Cancer Res..

[43] Zhang X., Yao J., Li X., Niu N., Liu Y., Hajek R.A., Peng G., Westin S., Sood A.K., Liu J. (2023). Targeting Polyploid Giant Cancer Cells Potentiates a Therapeutic Response and Overcomes Resistance to PARP Inhibitors in Ovarian Cancer. Sci. Adv..

[44] Pienta K.J., Hammarlund E.U., Axelrod R., Brown J.S., Amend S.R. (2020). Poly-Aneuploid Cancer Cells Promote Evolvability, Generating Lethal Cancer. Evol. Appl..

[45] Khan A., Zhang Y., Ma N., Shi J., Hou Y. (2024). NF-κB Role on Tumor Proliferation, Migration, Invasion and Immune Escape. Cancer Gene Ther..

[46] Mallin M.M., Rolle L.T.A., Schmidt M.J., Priyadarsini Nair S., Zurita A.J., Kuhn P., Hicks J., Pienta K.J., Amend S.R. (2025). Cells in the Polyaneuploid Cancer Cell State Are Prometastatic. Mol. Cancer Res..

[47] Perelli L., Carbone F., Zhang L., Huang J.K., Le C., Khan H., Citron F., Del Poggetto E., Gutschner T., Tomihara H. (2023). Interferon Signaling Promotes Tolerance to Chromosomal Instability during Metastatic Evolution in Renal Cancer. Nat. Cancer.

[48] Ablasser A., Chen Z.J. (2019). cGAS in Action: Expanding Roles in Immunity and Inflammation. Science.

[49] Garcia-Diaz A., Shin D.S., Moreno B.H., Saco J., Escuin-Ordinas H., Rodriguez G.A., Zaretsky J.M., Sun L., Hugo W., Wang X. (2017). Interferon Receptor Signaling Pathways Regulating PD-L1 and PD-L2 Expression. Cell Rep..

[50] Rumnieks F., Vainshelbaum N.M., Salmina K., Lazovska M., Kreismane M., Pjanova D., Zalums J., Vaivode K., Saliba D.R., Zayakin P. (2026). Resistance of BRAFV600E-Mutant Melanoma to Vemurafenib: A Senescence-Induced Swing from Differentiation to Blastulation Followed by Proliferation. Cancer Lett..

[51] Chen C., Chen J., Zhang Y., Zhang Q., Shi H. (2025). Senescence-Associated Secretory Phenotype in Lung Cancer: Remodeling the Tumor Microenvironment for Metastasis and Immune Suppression. Front. Oncol..

[52] Lin X., Kang K., Chen P., Zeng Z., Li G., Xiong W., Yi M., Xiang B. (2024). Regulatory Mechanisms of PD-1/PD-L1 in Cancers. Mol. Cancer.

[53] Liu D., Wang J., Wei Y., Wu H., Yuan S., Yang M., Sun L. (2025). Senescent Cancer Cells in Immune Surveillance and Evasion: Mechanisms and Therapeutic Implications. J. Leukoc. Biol..

[54] Liu P., Wang L., Yu H. (2024). Polyploid Giant Cancer Cells: Origin, Possible Pathways of Formation, Characteristics, and Mechanisms of Regulation. Front. Cell Dev. Biol..

[55] Niu N., Yao J., Bast R.C., Sood A.K., Liu J. (2021). IL-6 Promotes Drug Resistance through Formation of Polyploid Giant Cancer Cells and Stromal Fibroblast Reprogramming. Oncogenesis.

[56] Sikora E., Czarnecka-Herok J., Bojko A., Sunderland P. (2022). Therapy-Induced Polyploidization and Senescence: Coincidence or Interconnection?. Semin. Cancer Biol..

[57] Agudo J., Miao Y. (2025). Stemness in Solid Malignancies: Coping with Immune Attack. Nat. Rev. Cancer.

[58] Bayik D., Lathia J.D. (2021). Cancer Stem Cell-Immune Cell Crosstalk in Tumour Progression. Nat. Rev. Cancer.

[59] Erenpreisa J., Vainshelbaum N.M., Lazovska M., Karklins R., Salmina K., Zayakin P., Rumnieks F., Inashkina I., Pjanova D., Erenpreiss J. (2023). The Price of Human Evolution: Cancer-Testis Antigens, the Decline in Male Fertility and the Increase in Cancer. Int. J. Mol. Sci..

[60] Herbein G., El Baba R. (2024). Polyploid Giant Cancer Cells: A Distinctive Feature in the Transformation of Epithelial Cells by High-Risk Oncogenic HCMV Strains. Viruses.

[61] Liu J. (2020). The “Life Code”: A Theory That Unifies the Human Life Cycle and the Origin of Human Tumors. Semin. Cancer Biol..

[62] Liu J. (2018). The Dualistic Origin of Human Tumors. Semin. Cancer Biol..

[63] Salmina K., Vainshelbaum N.M., Kreishmane M., Inashkina I., Cragg M.S., Pjanova D., Erenpreisa J. (2023). The Role of Mitotic Slippage in Creating a “Female Pregnancy-like System” in a Single Polyploid Giant Cancer Cell. Int. J. Mol. Sci..

[64] Erenpreisa J., Salmina K., Huna A., Jackson T.R., Vazquez-Martin A., Cragg M.S. (2015). The “Virgin Birth”, Polyploidy, and the Origin of Cancer. Oncoscience.

[65] Illidge T.M., Cragg M.S., Fringes B., Olive P., Erenpreisa J.A. (2000). Polyploid Giant Cells Provide a Survival Mechanism for P53 Mutant Cells after DNA Damage. Cell Biol. Int..

[66] Chang W.H., Lai A.G. (2019). Aberrations in Notch-Hedgehog Signalling Reveal Cancer Stem Cells Harbouring Conserved Oncogenic Properties Associated with Hypoxia and Immunoevasion. Br. J. Cancer.

[67] Anatskaya O.V., Vinogradov A.E. (2024). Polyploidy Promotes Hypertranscription, Apoptosis Resistance, and Ciliogenesis in Cancer Cells and Mesenchymal Stem Cells of Various Origins: Comparative Transcriptome In Silico Study. Int. J. Mol. Sci..

[68] Erenpreisa J., Cragg M.S., Pontarotti P. (2008). Life-Cycle Features of Tumour Cells. Evolutionary Biology from Concept to Application.

[69] Ērenpreiss J.-O. (1993). Current Concepts of Malignant Growth. Part A: From a Normal Cell to Cancer.

[70] Behnam B., Fazilaty H., Ghadyani M., Fadavi P., Taghizadeh-Hesary F. (2023). Ciliated, Mitochondria-Rich Postmitotic Cells Are Immune-Privileged, and Mimic Immunosuppressive Microenvironment of Tumor-Initiating Stem Cells: From Molecular Anatomy to Molecular Pathway. Front. Biosci. (Landmark Ed.).

[71] Bowes A.L., Waise S., Lesluyes T., Butters T., English C., Yan H., Verfaillie A., Davies C., Chen J., Nye E. (2026). Profiling the Genomic Landscape and Evolutionary History of Polyploid Giant Cancer Cells in Undifferentiated Pleomorphic Sarcomas. Cancer Lett..

[72] Mirzayans R., Murray D. (2020). Intratumor Heterogeneity and Therapy Resistance: Contributions of Dormancy, Apoptosis Reversal (Anastasis) and Cell Fusion to Disease Recurrence. Int. J. Mol. Sci..

[73] Furth N., Aylon Y. (2017). The LATS1 and LATS2 Tumor Suppressors: Beyond the Hippo Pathway. Cell Death Differ..

[74] Jin S., Tong T., Fan W., Fan F., Antinore M.J., Zhu X., Mazzacurati L., Li X., Petrik K.L., Rajasekaran B. (2002). GADD45-Induced Cell Cycle G2-M Arrest Associates with Altered Subcellular Distribution of Cyclin B1 and Is Independent of P38 Kinase Activity. Oncogene.

[75] Perucca P., Cazzalini O., Madine M., Savio M., Laskey R.A., Vannini V., Prosperi E., Stivala L.A. (2009). Loss of P21 CDKN1A Impairs Entry to Quiescence and Activates a DNA Damage Response in Normal Fibroblasts Induced to Quiescence. Cell Cycle.

[76] Friedman A.D. (2009). Cell Cycle and Developmental Control of Hematopoiesis by Runx1. J. Cell Physiol..

[77] McConnell B.B., Yang V.W. (2010). Mammalian Krüppel-like Factors in Health and Diseases. Physiol. Rev..

[78] Mitra M., Batista S.L., Coller H.A. (2025). Transcription Factor Networks in Cellular Quiescence. Nat. Cell Biol..

[79] Santos-de-Frutos K., Djouder N. (2021). When Dormancy Fuels Tumour Relapse. Commun. Biol..

[80] Dhimolea E., de Matos Simoes R., Kansara D., Al’Khafaji A., Bouyssou J., Weng X., Sharma S., Raja J., Awate P., Shirasaki R. (2021). An Embryonic Diapause-like Adaptation with Suppressed Myc Activity Enables Tumor Treatment Persistence. Cancer Cell.

[81] Liu K. (2010). Role of Apoptosis Resistance in Immune Evasion and Metastasis of Colorectal Cancer. World J. Gastrointest. Oncol..

[82] Cao Y. (2023). Neural Induction Drives Body Axis Formation during Embryogenesis, but a Neural Induction-like Process Drives Tumorigenesis in Postnatal Animals. Front. Cell Dev. Biol..

[83] Toledano S., Neufeld G. (2023). Plexins as Regulators of Cancer Cell Proliferation, Migration, and Invasivity. Cancers.

[84] Tajada S., Villalobos C. (2020). Calcium Permeable Channels in Cancer Hallmarks. Front. Pharmacol..

[85] Nandakumar S., Rozich E., Buttitta L. (2021). Cell Cycle Re-Entry in the Nervous System: From Polyploidy to Neurodegeneration. Front. Cell Dev. Biol..

[86] Bezprozvanny I. (2026). Calcium Signaling and Pathogenesis of Neurodegenerative Disorders: Potential Therapeutic Opportunities. Cold Spring Harb. Perspect. Biol..

[87] Oshkolova A.A., Grekhnev D.A., Kruchinina A.A., Belikova L.D., Volovikov E.A., Lebedeva O.S., Bogomazova A.N., Vigont V.A., Lagarkova M.A., Kaznacheyeva E.V. (2024). Comparison of the Calcium Signaling Alterations in GABA-Ergic Medium Spiny Neurons Produced from iPSCs of Different Origins. Biochimie.

[88] Skobeleva K., Wang G., Kaznacheyeva E. (2025). STIM Proteins: The Gas and Brake of Calcium Entry in Neurons. Neurosci. Bull..

[89] Popugaeva E., Chernyuk D., Bezprozvanny I. (2020). Reversal of Calcium Dysregulation as Potential Approach for Treating Alzheimer’s Disease. Curr. Alzheimer Res..

[90] Sileo P., Simonin C., Melnyk P., Chartier-Harlin M.-C., Cotelle P. (2022). Crosstalk between the Hippo Pathway and the Wnt Pathway in Huntington’s Disease and Other Neurodegenerative Disorders. Cells.

[91] Quinton R.J., DiDomizio A., Vittoria M.A., Kotýnková K., Ticas C.J., Patel S., Koga Y., Vakhshoorzadeh J., Hermance N., Kuroda T.S. (2021). Whole-Genome Doubling Confers Unique Genetic Vulnerabilities on Tumour Cells. Nature.

[92] Lu L., Zha Z., Zhang P., Wang P., Liu X., Fang X., Weng C., Li B., Mao H., Wang L. (2022). Neuron-Specific Enolase Promotes Stem Cell-like Characteristics of Small-Cell Lung Cancer by Downregulating NBL1 and Activating the BMP2/Smad/ID1 Pathway. Oncogenesis.

[93] Zhang M., Liu Y., Shi L., Fang L., Xu L., Cao Y. (2022). Neural Stemness Unifies Cell Tumorigenicity and Pluripotent Differentiation Potential. J. Biol. Chem..

[94] Roig Adam A., Martínez-López J.A., van der Spek S.J.F., SYNGO consortium, Sullivan P.F., Smit A.B., Verhage M., Hjerling-Leffler J. (2023). Transcriptional Diversity in Specific Synaptic Gene Sets Discriminates Cortical Neuronal Identity. Biol. Direct.

[95] Kulatunga D.C.M., Kim M.K. (2026). Context-Tuned Strategies for Marker Selection Precision in Neuronal Studies. Front. Neurosci..

[96] Huang P., Wu R., Yang Z., Li Y., Fei F., Yu Y. (2025). Polyploid Giant Cancer Cells and Tumor Budding: Translation from Basic Research to Clinical Application. Front. Oncol..

[97] Hanahan D. (2026). Hallmarks of Cancer-Then and Now, and Beyond. Cell.

[98] Casotti M.C., Meira D.D., Zetum A.S.S., Araújo B.C.d., Silva D.R.C.d., Santos E.d.V.W.D., Garcia F.M., Paula F.d., Santana G.M., Louro L.S. (2023). Computational Biology Helps Understand How Polyploid Giant Cancer Cells Drive Tumor Success. Genes.

[99] Tarumi W., Murai K., Nakahata Y., Masui K. (2026). The Paradox of Senescence in Glioblastoma: SASP as an Emerging Cancer Hallmark. Cancers.

[100] Xavier F.C.A., Silva J.C., Rodini C.O., Rodrigues M.F.S.D. (2022). Mechanisms of Immune Evasion by Head and Neck Cancer Stem Cells. Front. Oral Health.

[101] Zhou M., Niu H., Cui D., Xu M., Li J., Huang G., Zhou M., Xiong C., Liu Y., Xu X. (2026). Cancer Stem Cell-Driven Drug Resistance in Colorectal Carcinoma: Molecular Aspects and Therapeutic Potentials. Mol. Cancer.

[102] Kortleve D., Coelho R.M.L., Hammerl D., Debets R. (2022). Cancer Germline Antigens and Tumor-Agnostic CD8+ T Cell Evasion. Trends Immunol..

[103] Ma W., Wei L., Jin L., Ma Q., Zhang T., Zhao Y., Hua J., Zhang Y., Wei W., Ding N. (2024). YAP/Aurora A-Mediated Ciliogenesis Regulates Ionizing Radiation-Induced Senescence via Hedgehog Pathway in Tumor Cells. Biochim. Biophys. Acta Mol. Basis Dis..

[104] De Blander H., Morel A.-P., Senaratne A.P., Ouzounova M., Puisieux A. (2021). Cellular Plasticity: A Route to Senescence Exit and Tumorigenesis. Cancers.

[105] Menendez J.A., Alarcón T. (2017). Senescence-Inflammatory Regulation of Reparative Cellular Reprogramming in Aging and Cancer. Front. Cell Dev. Biol..

[106] Yu X., Xu J. (2024). TWIST1 Drives Cytotoxic CD8+ T-Cell Exhaustion through Transcriptional Activation of CD274 (PD-L1) Expression in Breast Cancer Cells. Cancers.

[107] Wu J., Hu W., Yang W., Long Y., Chen K., Li F., Ma X., Li X. (2024). Knockdown of SQLE Promotes CD8+ T Cell Infiltration in the Tumor Microenvironment. Cell Signal..

[108] Müller T.R., Gao Y., Wu J., Ribeiro O., Chen P., Bergman P., Blennow O., Hansson L., Mielke S., Nowak P. (2024). Memory T Cells Effectively Recognize the SARS-CoV-2 Hypermutated BA.2.86 Variant. Cell Host Microbe.

[109] Wang H.-F., Wang S.-S., Huang M.-C., Liang X.-H., Tang Y.-J., Tang Y.-L. (2019). Targeting Immune-Mediated Dormancy: A Promising Treatment of Cancer. Front. Oncol..

[110] Liu J. (2022). Giant Cells: Linking McClintock’s Heredity to Early Embryogenesis and Tumor Origin throughout Millennia of Evolution on Earth. Semin. Cancer Biol..

[111] Bloomer H., Dame H.B., Parker S.R., Oudin M.J. (2025). Neuronal Mimicry in Tumors: Lessons from Neuroscience to Tackle Cancer. Cancer Metastasis Rev..

[112] Keough M.B., Monje M. (2022). Neural Signaling in Cancer. Annu. Rev. Neurosci..

[113] White-Gilbertson S., Lu P., Saatci O., Sahin O., Delaney J.R., Ogretmen B., Voelkel-Johnson C. (2024). Transcriptome Analysis of Polyploid Giant Cancer Cells and Their Progeny Reveals a Functional Role for P21 in Polyploidization and Depolyploidization. J. Biol. Chem..

[114] Shomar A., Barak O., Brenner N. (2022). Cancer Progression as a Learning Process. iScience.

[115] Anatskaya O.V., Runov A.L., Ponomartsev S.V., Vonsky M.S., Elmuratov A.U., Vinogradov A.E. (2023). Long-Term Transcriptomic Changes and Cardiomyocyte Hyperpolyploidy after Lactose Intolerance in Neonatal Rats. Int. J. Mol. Sci..

[116] Anatskaya O.V., Vinogradov A.E. (2010). Somatic Polyploidy Promotes Cell Function under Stress and Energy Depletion: Evidence from Tissue-Specific Mammal Transcriptome. Funct. Integr. Genom..

[117] Anatskaya O.V., Vinogradov A.E. (2007). Genome Multiplication as Adaptation to Tissue Survival: Evidence from Gene Expression in Mammalian Heart and Liver. Genomics.

[118] Vazquez-Martin A., Anatskaya O.V., Giuliani A., Erenpreisa J., Huang S., Salmina K., Inashkina I., Huna A., Nikolsky N.N., Vinogradov A.E. (2016). Somatic Polyploidy Is Associated with the Upregulation of C-MYC Interacting Genes and EMT-like Signature. Oncotarget.

[119] Foidart P., Li Z., Cai X., Seehawer M., Brown D.D., Tawawalla A., Baldominos P., Parvin S., Nishida J., Rojas-Jimenez E. (2026). Whole-Genome Doubling Drives Immune Evasion by Silencing Antigen Presentation. Cancer Cell.

[120] Kasperski A., Heng H.H. (2024). The Spiral Model of Evolution: Stable Life Forms of Organisms and Unstable Life Forms of Cancers. Int. J. Mol. Sci..

[121] Gemble S., Wardenaar R., Keuper K., Srivastava N., Nano M., Macé A.-S., Tijhuis A.E., Bernhard S.V., Spierings D.C.J., Simon A. (2022). Genetic Instability from a Single S Phase after Whole-Genome Duplication. Nature.

[122] Kciuk M., Kołat D., Kałuzińska-Kołat Ż., Gawrysiak M., Drozda R., Celik I., Kontek R. (2023). PD-1/PD-L1 and DNA Damage Response in Cancer. Cells.

[123] Sato H., Niimi A., Yasuhara T., Permata T.B.M., Hagiwara Y., Isono M., Nuryadi E., Sekine R., Oike T., Kakoti S. (2017). DNA Double-Strand Break Repair Pathway Regulates PD-L1 Expression in Cancer Cells. Nat. Commun..

[124] Wang N.-H., Lei Z., Yang H.-N., Tang Z., Yang M.-Q., Wang Y., Sui J.-D., Wu Y.-Z. (2022). Radiation-Induced PD-L1 Expression in Tumor and Its Microenvironment Facilitates Cancer-Immune Escape: A Narrative Review. Ann. Transl. Med..

[125] Baram T., Rubinstein-Achiasaf L., Ben-Yaakov H., Ben-Baruch A. (2020). Inflammation-Driven Breast Tumor Cell Plasticity: Stemness/EMT, Therapy Resistance and Dormancy. Front. Oncol..

[126] Heng J., Heng H.H. (2023). Karyotype as Code of Codes: An Inheritance Platform to Shape the Pattern and Scale of Evolution. Biosystems.

[127] Heng J., Heng H.H. (2022). Genome Chaos: Creating New Genomic Information Essential for Cancer Macroevolution. Semin. Cancer Biol..

[128] Heng J., Heng H.H. (2021). Two-Phased Evolution: Genome Chaos-Mediated Information Creation and Maintenance. Prog. Biophys. Mol. Biol..

[129] Li Q., Wennborg A., Aurell E., Dekel E., Zou J.-Z., Xu Y., Huang S., Ernberg I. (2016). Dynamics inside the Cancer Cell Attractor Reveal Cell Heterogeneity, Limits of Stability, and Escape. Proc. Natl. Acad. Sci. USA.

[130] Mojtahedi M., Skupin A., Zhou J., Castaño I.G., Leong-Quong R.Y.Y., Chang H., Trachana K., Giuliani A., Huang S. (2016). Cell Fate Decision as High-Dimensional Critical State Transition. PLoS Biol..

[131] Clough E., Barrett T., Wilhite S.E., Ledoux P., Evangelista C., Kim I.F., Tomashevsky M., Marshall K.A., Phillippy K.H., Sherman P.M. (2024). NCBI GEO: Archive for Gene Expression and Epigenomics Data Sets: 23-Year Update. Nucleic Acids Res..

[132] White-Gilbertson S., Lu P., Esobi I., Echesabal-Chen J., Mulholland P.J., Gooz M., Ogretmen B., Stamatikos A., Voelkel-Johnson C. (2022). Polyploid Giant Cancer Cells Are Dependent on Cholesterol for Progeny Formation through Amitotic Division. Sci. Rep..

[133] Rau A., Marot G., Jaffrézic F. (2014). Differential Meta-Analysis of RNA-Seq Data from Multiple Studies. BMC Bioinform..

[134] Sweeney T.E., Haynes W.A., Vallania F., Ioannidis J.P., Khatri P. (2017). Methods to Increase Reproducibility in Differential Gene Expression via Meta-Analysis. Nucleic Acids Res..

[135] Soneson C., Delorenzi M. (2013). A Comparison of Methods for Differential Expression Analysis of RNA-Seq Data. BMC Bioinform..

[136] Jabato F.M., Córdoba-Caballero J., Rojano E., Romá-Mateo C., Sanz P., Pérez B., Gallego D., Seoane P., Ranea J.A.G., Perkins J.R. (2021). Gene Expression Analysis Method Integration and Co-Expression Module Detection Applied to Rare Glucide Metabolism Disorders Using ExpHunterSuite. Sci. Rep..

[137] Kristiansson E., Österlund T., Gunnarsson L., Arne G., Larsson D.G.J., Nerman O. (2013). A Novel Method for Cross-Species Gene Expression Analysis. BMC Bioinform..

[138] Nithya C., Kiran M., Nagarajaram H.A. (2023). Dissection of Hubs and Bottlenecks in a Protein-Protein Interaction Network. Comput. Biol. Chem..

[139] Pang E., Hao Y., Sun Y., Lin K. (2016). Differential Variation Patterns between Hubs and Bottlenecks in Human Protein-Protein Interaction Networks. BMC Evol. Biol..

[140] Shantaraman A., Dammer E.B., Ugochukwu O., Duong D.M., Yin L., Carter E.K., Gearing M., Chen-Plotkin A., Lee E.B., Trojanowski J.Q. (2024). Correction: Network Proteomics of the Lewy Body Dementia Brain Reveals Presynaptic Signatures Distinct from Alzheimer’s Disease. Mol. Neurodegener..

[141] He X., Zhang J. (2006). Why Do Hubs Tend to Be Essential in Protein Networks?. PLoS Genet..

[142] Zotenko E., Mestre J., O’Leary D.P., Przytycka T.M. (2008). Why Do Hubs in the Yeast Protein Interaction Network Tend to Be Essential: Reexamining the Connection between the Network Topology and Essentiality. PLoS Comput. Biol..

[143] Zhou Y., Zhou B., Pache L., Chang M., Khodabakhshi A.H., Tanaseichuk O., Benner C., Chanda S.K. (2019). Metascape Provides a Biologist-Oriented Resource for the Analysis of Systems-Level Datasets. Nat. Commun..

[144] Szklarczyk D., Nastou K., Koutrouli M., Kirsch R., Mehryary F., Hachilif R., Hu D., Peluso M.E., Huang Q., Fang T. (2025). The STRING Database in 2025: Protein Networks with Directionality of Regulation. Nucleic Acids Res..

