# Supplementary material for: Inflamed Yet Immune-Evasive? A Transcriptomic Meta-Analysis Identifies Conserved Inflammatory, Developmental, and Neuronal Signatures Associated with Polyploid Giant Cancer Cells
Source: Int J Mol Sci. 2026 Jul 26;27(15):6671. doi: 10.3390/ijms27156671 (PMC13466468; doi:10.3390/ijms27156671)
Supplement: Supplementary file 1 [file ijms-27-06671-s001.zip › Supplementary figures S1-S4_Revised_27.07.2026.pdf]

Supplementary Figures

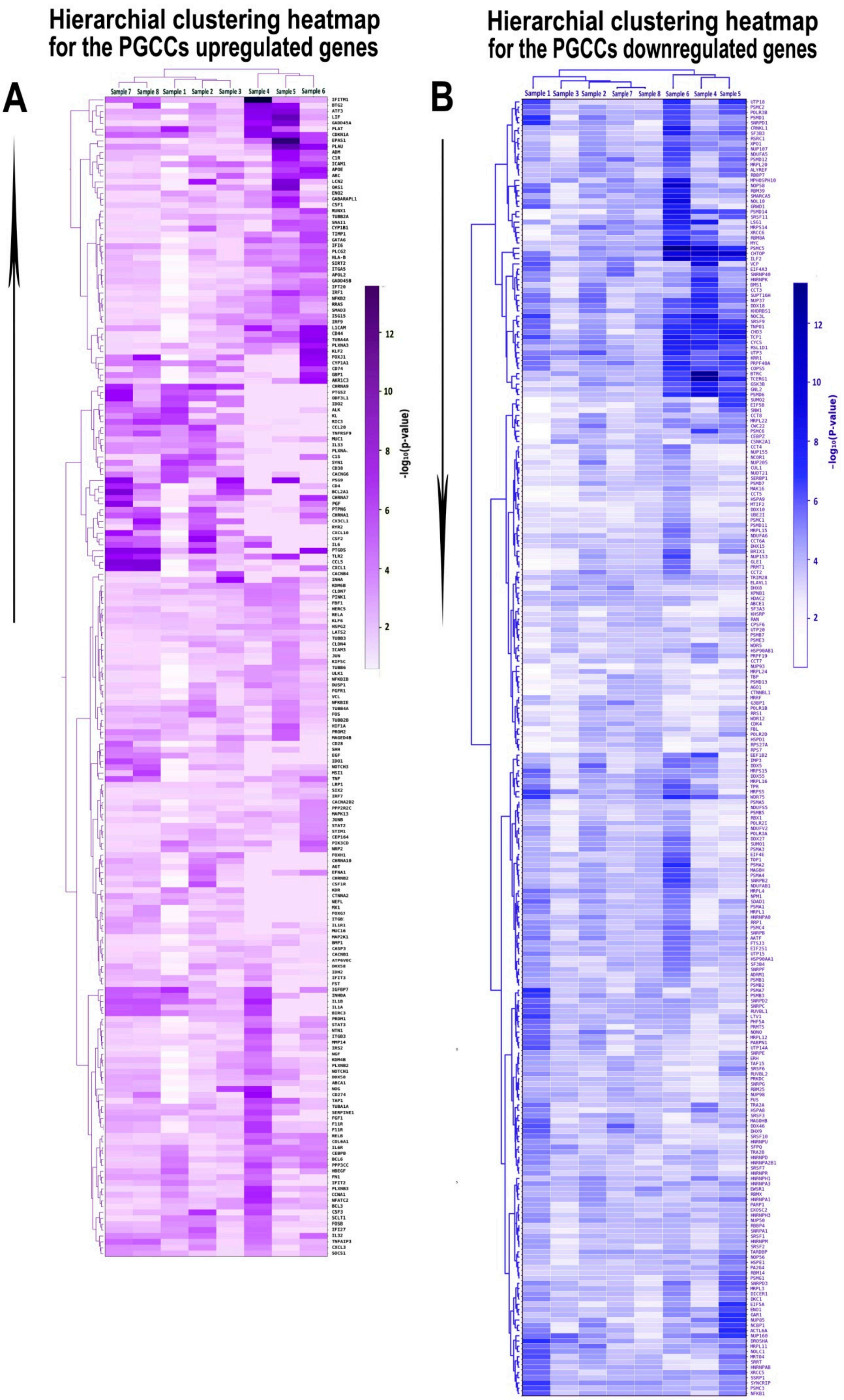
Hierarchial clustering heatmap
for the PGCCs upregulated genes
A
Sample 7
Sample 8
Sample 1
Sample 2
Sample 3
Sample 4
Sample 5
Sample 6
-log10(p-value)
Hierarchial clustering heatmap
for the PGCCs downregulated genes
B
Sample 1
Sample 3
Sample 2
Sample 7
Sample 8
Sample 6
Sample 4
Sample 5
-log10(P-value)

Supplementary Figure S1. Heatmaps of PGCC-associated genes showing the statistical significance of differential gene expression across independent datasets.

(A) Hierarchical clustering heatmap of genes upregulated in PGCCs relative to their diploid counterparts across eight independent datasets. Color intensity represents the statistical significance of differential expression expressed as −log10(*p*-value). Rows correspond to genes and columns correspond to individual PGCC datasets. Samples are arranged according to hierarchical clustering based on similarity in differential-expression significance profiles. **(B)** Hierarchical clustering heatmap of genes downregulated in PGCCs relative to diploid cancer cells. Color intensity represents the statistical significance of differential expression expressed as −log10(*p*-value). Rows correspond to genes and columns correspond to individual PGCC datasets. Samples are arranged according to hierarchical clustering.

Sample coding: (1) PGCCs generated from the prostate cancer cell line PPC1 (2) PGCCs derived from the ovarian cancer cell line HEY following paclitaxel treatment; (3) PGCCs derived from the ovarian cancer cell line SKOV3 following paclitaxel treatment; (4) PGCCs generated from the ovarian cancer cell line HEY following treatment with the PARP inhibitor olaparib; (5) PGCCs generated from the breast cancer cell line MCF7 following olaparib treatment; (6) PGCCs generated from organoid 2414 following olaparib treatment; (7) PGCCs derived from irradiated PPC1 prostate cancer cells; and (8) PGCCs derived from irradiated PPC1 cells treated with the acid ceramidase inhibitor LCL521.

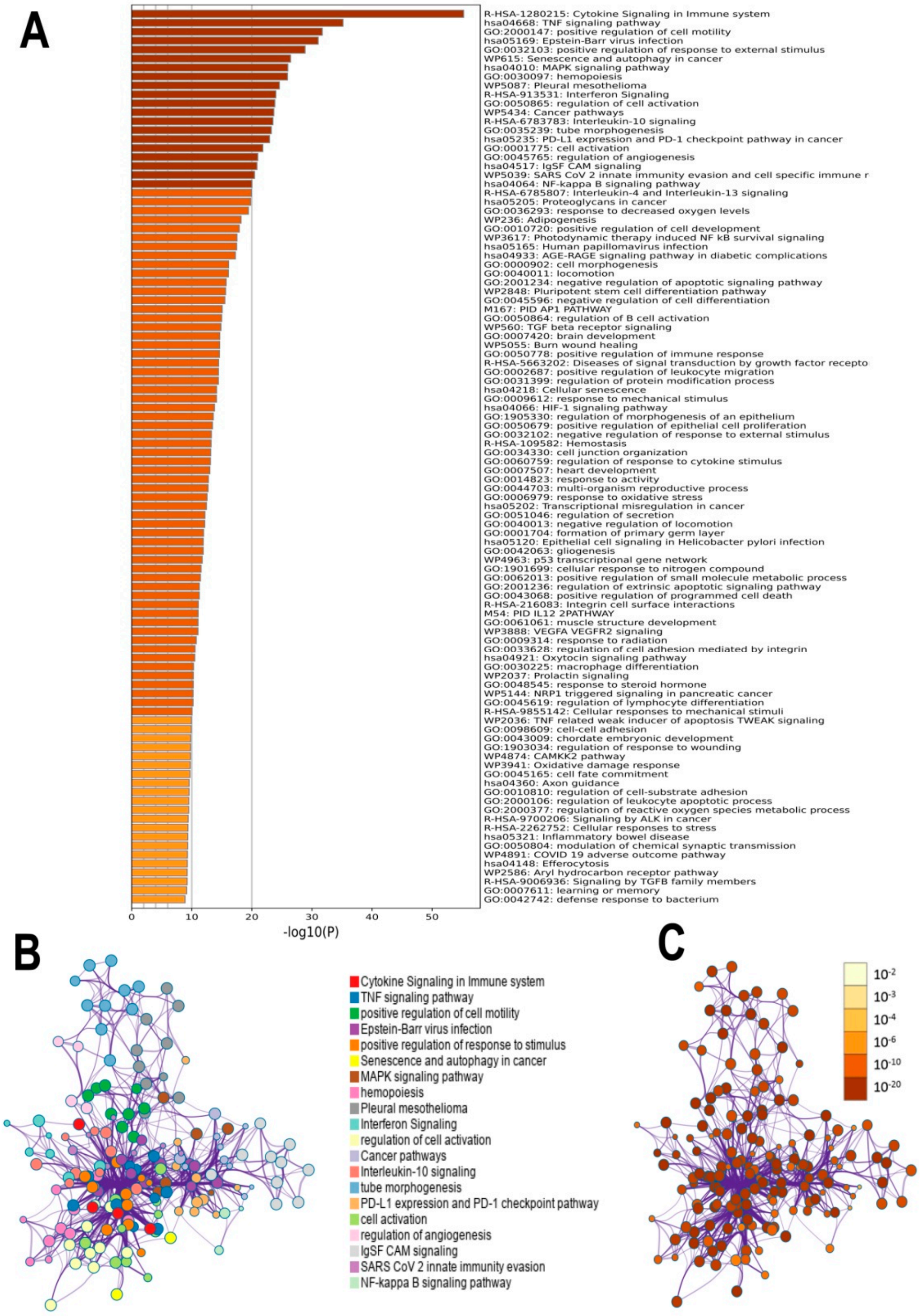

Supplementary Figure S2. Enrichment of PGCC-induced genes in GO biological processes and KEGG, REACTOME and WIKI signaling pathways **A)** Bar graph of enriched terms across PGCCs upregulated genes colored by ***p***-value. The statistical significance of enrichment is shown on the X-axis (−log10(p)) **B**) Network of enriched terms colored by gene module ID, where nodes that share the same cluster ID are typically close to each other. **C)** Network of enriched terms colored by ***p***-value, where gene modules containing more genes tend to have more significant ***p***-value.

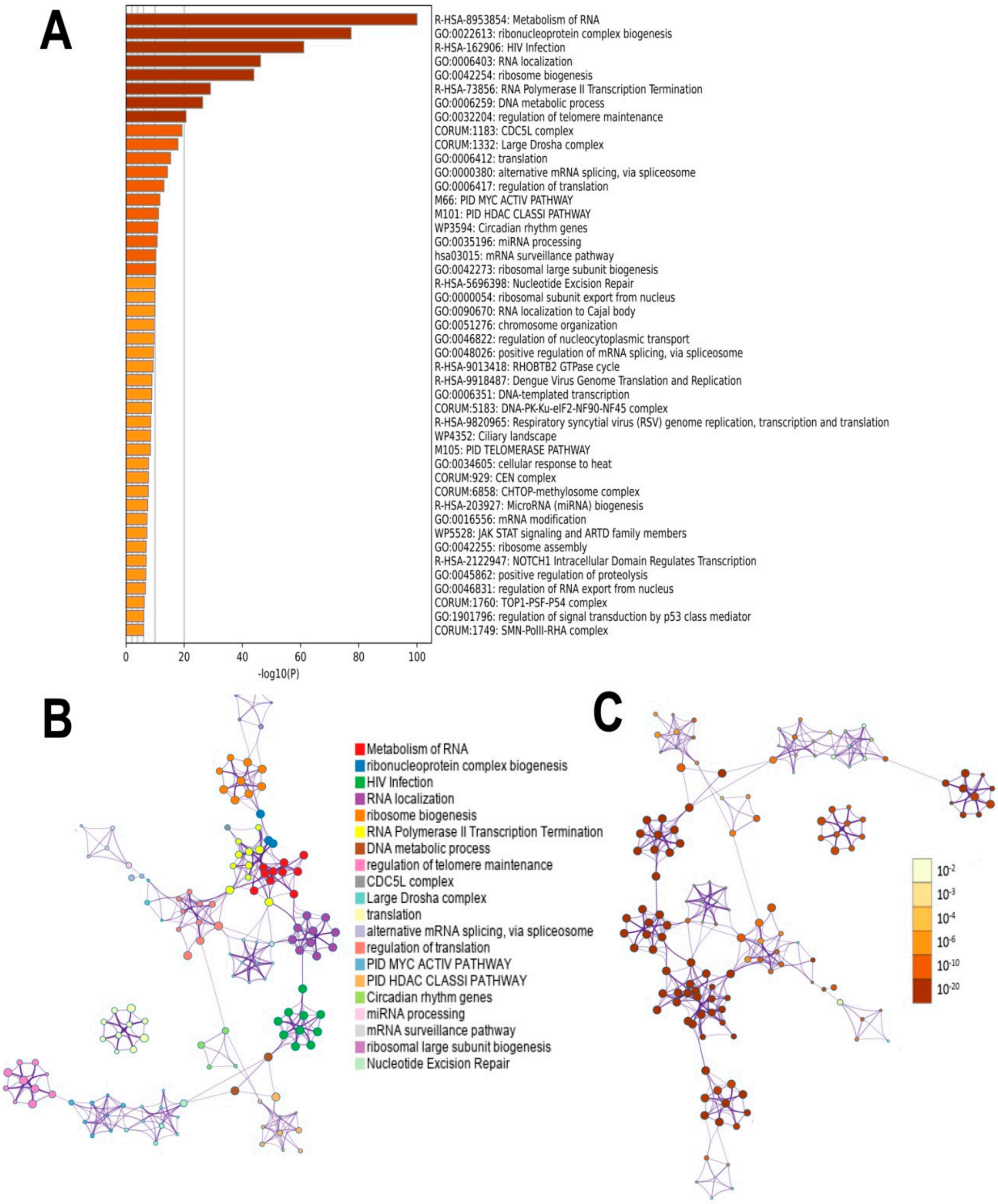

Supplementary Figure S3. Enrichment of PGCC-inhibited genes in GO biological processes and KEGG, REACTOME and WIKI signaling pathways **A)** Bar graph of enriched terms across PGCCs downregulated genes colored by ***p*-**value. The statistical significance of enrichment is shown on the X-axis (−log10(p)) **B**) Network of enriched terms colored by gene module ID, where nodes that share the same cluster ID are typically close to each other. **C)** Network of enriched terms colored by ***p*-**value, where gene modules containing more genes tend to have more significant ***p***-value.

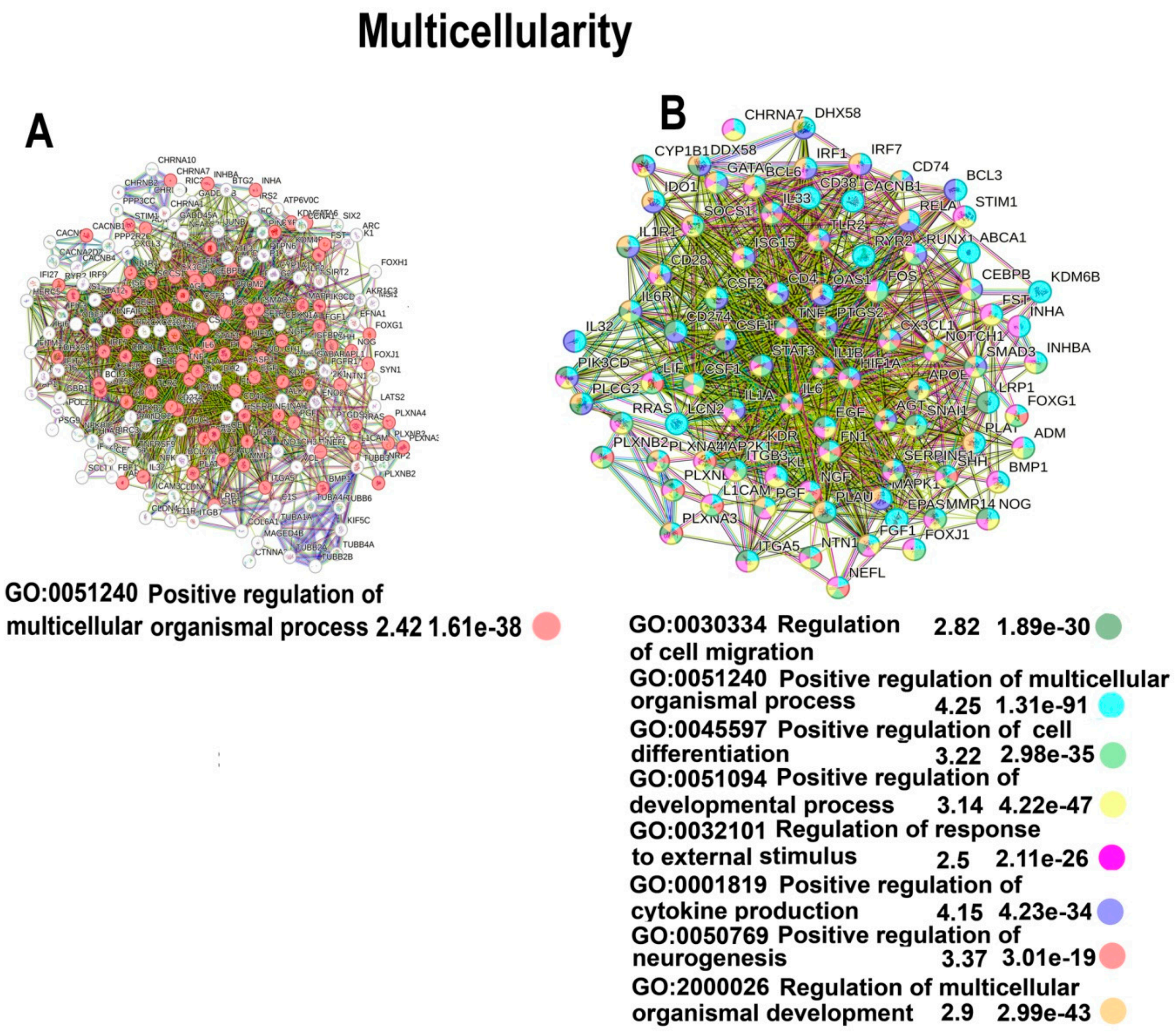

Supplemmentary Figure S4. Multicellularity-associated gene networks in polyploid giant cancer cells (PGCCs). Network analysis of genes linked to multicellular organization and developmental programs reveals extensive activation of multicellularity-related signaling in PGCCs. **(A)** Protein–protein interaction (PPI) network constructed from genes associated with the Gene Ontology (GO) term "*Positive regulation of multicellular organismal process*" (GO:0051240). The network forms a highly interconnected module enriched in regulators of cell communication, adhesion, developmental signaling, and tissue organization, indicating coordinated activation of multicellular traits. Node colors correspond to pathway membership, while edges represent known and predicted functional associations.

(B) Functional enrichment and interaction network of key multicellularity-associated genes. The network is enriched in biological processes related to *positive regulation of multicellular organismal process*, *regulation of multicellular organismal development*, *positive regulation of developmental process*, *positive regulation of cell differentiation*, *regulation of cell migration*, *positive regulation of cytokine production*, *regulation of response to external stimulus*, and *positive regulation of neurogenesis*. Central hub genes include inflammatory mediators (IL1A, IL1B, TNF, CSF1, CSF2), developmental regulators (NOTCH1, BMP1, SMAD3, FOXJ1), extracellular matrix and adhesion molecules (FN1, ITGA5, ICAM1), and growth factor signaling components (EGF, KDR, PGF, NGF). Together, these interconnected modules suggest that

PGCCs activate an evolutionarily conserved multicellular program characterized by enhanced cellular communication, environmental responsiveness, developmental plasticity, collective behavior, and tissue remodeling capacity, supporting adaptation, survival, and tumor progression under stress conditions.

Data were generated using STRING-based protein interaction analysis and Gene Ontology enrichment. Numbers indicate enrichment scores and adjusted *P*-values for the corresponding biological processes.
